# Robotic Gastrointestinal Surgery Compared to Conventional Approaches: An Umbrella Review of Clinical and Economic Outcomes

**DOI:** 10.3390/jcm14238555

**Published:** 2025-12-02

**Authors:** Seung Hyun Rho, Jeonghyun Lee, Jun Suh Lee

**Affiliations:** 1 Department of Medicine, Yonsei University College of Medicine, Seoul 03722, Republic of Korea; 2 Department of Medicine, Catholic University College of Medicine, Seoul 06591, Republic of Korea; 3 Department of Surgery, Bucheon Sejong Hospital, Bucheon 14754, Republic of Korea

**Keywords:** robotic surgery, gastrointestinal procedures, surgical outcome, cost-benefit analysis, minimally invasive surgical procedures

## Abstract

**Background/Objectives:** Robotic-assisted surgery (RAS) has emerged as a technological advancement in gastrointestinal (GI) procedures, addressing limitations of conventional laparoscopy through enhanced dexterity, three-dimensional visualization, and ergonomic improvements. While its clinical use is expanding, the comparative benefits and cost-effectiveness of RAS across different GI domains remain unclear. **Methods:** An umbrella review was conducted to evaluate RAS across six GI domains: esophageal, gastric, liver, biliary, pancreatic, and colorectal. A systematic literature search of PubMed was performed in April 2025, yielding 8961 articles. Reviews published in English since 2018 and comparing RAS with laparoscopic or open approaches in human GI surgery were eligible. A total of 250 articles met the inclusion criteria. Data on technical feasibility, clinical outcomes, and cost-effectiveness were extracted. Methodological quality was appraised using the AMSTAR 2 checklist. Results were synthesized narratively. The study was supported by the National Research Foundation of Korea grant, and the protocol was registered in PROSPERO (CRD420251042541). **Results:** RAS demonstrated domain-specific advantages. Esophageal and gastric surgeries benefited from enhanced precision and lymphadenectomy, while long-term outcomes were comparable to laparoscopy. Robotic liver and biliary surgeries offered technical advantages in complex cases, but evidence was limited. The most significant clinical benefits were observed in pancreatic and colorectal procedures, in which RAS reduced conversion rates and improved short-term outcomes in anatomically challenging scenarios. Cost-effectiveness was generally unfavorable but showed improvement in high-volume centers due to reduced complications and shorter hospital stays. **Conclusions:** Robotic assistance provides the most consistent clinical benefit in pancreatic and colorectal surgery, especially for complex, high-risk cases. While high procedural costs remain a barrier, selective use of RAS in appropriate settings may yield improved outcomes. These findings support the need for ongoing evaluation of cost-effectiveness and long-term results to guide evidence-based integration of robotics into GI surgery.

## 1. Introduction

Minimally invasive surgery (MIS) has transformed gastrointestinal (GI) surgery by reducing morbidity, shortening hospital stays, and improving recovery. However, conventional laparoscopy is limited by two-dimensional visualization, restricted instrument movement, and ergonomic strain on surgeons [1]. Robotic-assisted surgery (RAS), developed to overcome these limitations, offers enhanced dexterity, three-dimensional imaging, and improved ergonomics, facilitating complex GI procedures in confined anatomical spaces [2].

While robotic surgery was initially dominated by urological and gynecologic applications, recent trends show a rapid expansion into GI procedures. Gastric, liver, pancreatic and colorectal surgeries now represent a substantial and growing proportion of robotic cases worldwide, particularly in cancer-focused institutions. Since receiving FDA approval, robotic systems have been increasingly adopted in GI oncology.

More recently, the single-port (SP) system has allowed all instruments to be deployed through a single 2.5 cm incision using a specialized cannula, improving access in confined anatomical spaces such as the deep pelvis or retroperitoneum [3,4].

Although da Vinci remains the most widely used platform, other robotic systems, such as Senhance, Hugo, and Versius, have also emerged, expanding the landscape of robotic GI surgery. Evidence from multicenter reviews and meta-analyses suggests that robotic surgery leads to reduced conversion rates, blood loss, and postoperative complications compared to laparoscopic and open approaches [5].

Despite these clinical benefits, the high cost of robotic platforms remains controversial [6]. While some argue these expenses are justified by better outcomes and shorter hospital stays [5], others contend that current evidence is insufficient to justify widespread adoption [7]. This umbrella review compares robotic and conventional approaches across six GI domains—esophageal, gastric, liver, biliary, pancreatic, and colorectal—evaluating clinical outcomes and cost-effectiveness with the ultimate goal of identifying which procedures derive the greatest benefit from robotic assistance. In the existing literature, robotic surgery has been compared to both laparoscopic and open approaches, often within the same review. Because umbrella reviews synthesize data from previously published systematic reviews and meta-analyses, the comparator definitions follow those used in the included reviews. Therefore, in this study, the term ‘conventional approaches’ refers to laparoscopic and/or open surgery, depending on the comparator used in each individual review. The heterogeneity of comparators is acknowledged and discussed as an intrinsic methodological limitation of umbrella reviews.

## 2. Materials and Methods

### 2.1. Search Strategy

A comprehensive literature search was conducted in the PubMed (12 April 2025) database. Gastrointestinal procedures were categorized into six domains: esophageal, gastric, liver, biliary, pancreatic, and colorectal surgery. For each domain, relevant studies were identified using combinations of search terms related to robotic surgery, conventional surgical approaches, and domain-specific procedures; the full search strategies are provided in Supplementary Table S1. This umbrella review protocol was registered in PROSPERO (No. CRD420251042541) and is available at: https://www.crd.york.ac.uk/PROSPERO/view/CRD (accessed on 24 November 2025). The registered protocol outlines the objectives, eligibility criteria, search strategy, and planned data synthesis methods of this review.

The literature search was performed exclusively in PubMed. For each gastrointestinal domain, we used predefined keyword combinations including terms such as ‘robotic surgery’, ‘laparoscopic’, ‘open’, ‘esophagectomy’, ‘gastrectomy’, ‘hepatectomy’, ‘biliary surgery’, ‘pancreatectomy’, and ‘colorectal resection.’ Detailed domain-specific search strings (e.g., “robotic AND esophagectomy”, “robotic gastrectomy AND laparoscopy”, “robotic hepatectomy AND open”) are provided in Supplementary Table S1.

### 2.2. Eligibility Criteria

Articles published after January 2018 were selected for this study. During our systematic search, we observed a substantial increase in publications related to robotic gastrointestinal surgery beginning in 2018. To ensure clinical relevance and capture recent advancements in surgical robotics, we limited inclusion to articles published within this timeframe. Eligible study types included meta-analyses, systematic reviews, and narrative reviews. Only human studies published in English were included. We selected reviews that evaluated the clinical performance of robotic surgery compared to conventional (laparoscopic or open) approaches. Reviews covering multiple or unrelated surgical sites were excluded.

### 2.3. Screening and Data Extraction

Two independent researchers screened all records for relevance based on titles and abstracts. Full-text articles of potentially eligible studies were assessed against the predefined inclusion and exclusion criteria. Any discrepancies between reviewers were resolved through discussion and consensus. For all eligible publications, data were manually extracted into standardized tables, including details on surgical domain, procedure type, surgical approach, and key findings. A full quality control check of the extracted data was conducted by two researchers to ensure accuracy and completeness.

Comparators were classified as ‘conventional approaches’, a term that reflects the definitions used in the included reviews. These comparators included laparoscopic and/or open surgery depending on the scope of each review. Because umbrella reviews synthesize previously published reviews rather than re-analyzing primary data, comparator heterogeneity could not be standardized across domains.

### 2.4. Methodological Quality Assessment

To assess the methodological rigor of the included reviews, the AMSTAR 2 (A Measurement Tool to Assess Systematic Reviews 2) checklist was applied. This tool evaluates the quality of systematic reviews based on 16 critical and non-critical domains, and assigns an overall confidence rating (high, moderate, low, or critically low) in the validity of each review’s findings. All assessments were performed independently by two reviewers, and discrepancies were resolved by consensus.

To assess the degree of overlap among included systematic reviews, we calculated the Corrected Covered Area (CCA), a validated metric quantifying the frequency of shared primary studies across reviews [8]. The CCA quantifies the percentage of overlapping primary studies across reviews, accounting for both the number of reviews and the number of unique studies. A CCA of 0–5% was interpreted as slight overlap, 6–10% as moderate, 11–15% as high, and >15% as very high overlap. This review followed PRISMA 2020 guidelines; the completed checklist is provided in Supplementary Tables S2 and S3.

## 3. Results

### 3.1. Study Selection

**Figure 1:**
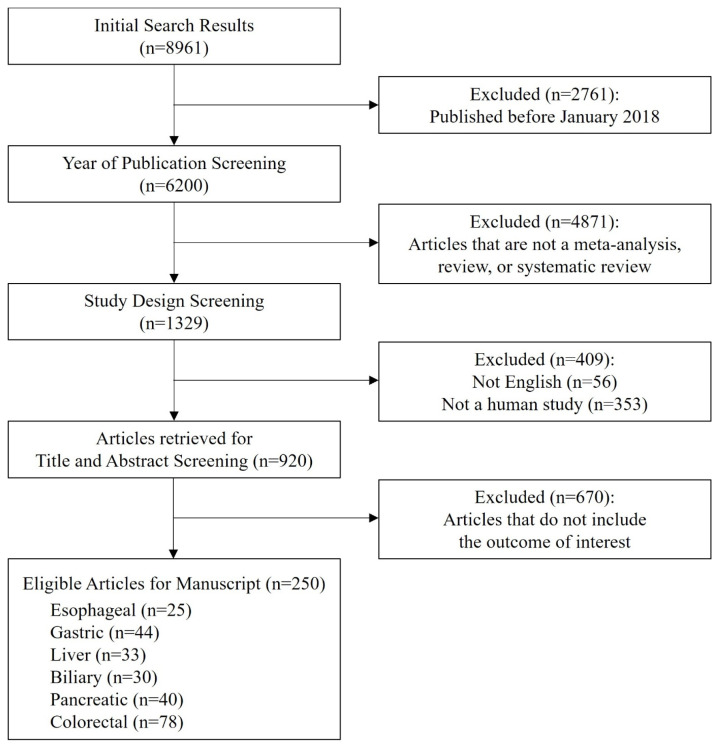
Flow diagram of the study selection process.

A total of 8961 articles were initially identified through the systematic literature search. After restricting the publication period to studies published between 2018 and 2025, 6200 articles remained. Following the application of eligibility criteria based on article type, 4871 studies were excluded. An additional 56 non-English articles were removed. Subsequently, 920 articles involving human subjects were selected for further screening. After reviewing titles and abstracts, 670 articles were excluded due to irrelevance to the study topic, leaving 250 articles selected for full-text review. Of these, 25 studies focused on esophageal surgery [5,9,10,11,12,13,14,15,16,17,18,19,20,21,22,23,24,25,26,27,28,29,30,31,32], 44 on gastric surgery [33,34,35,36,37,38,39,40,41,42,43,44,45,46,47,48,49,50,51,52,53,54,55,56,57,58,59,60,61,62,63,64,65,66,67,68,69,70,71,72,73,74,75,76], 33 on liver surgery [77,78,79,80,81,82,83,84,85,86,87,88,89,90,91,92,93,94,95,96,97,98,99,100,101,102,103,104,105,106,107,108,109], 30 on biliary surgery [110,111,112,113,114,115,116,117,118,119,120,121,122,123,124,125,126,127,128,129,130,131,132,133,134,135,136,137,138,139], 40 on pancreatic surgery [6,140,141,142,143,144,145,146,147,148,149,150,151,152,153,154,155,156,157,158,159,160,161,162,163,164,165,166,167,168,169,170,171,172,173,174,175,176,177], and 78 on colorectal surgery [178,179,180,181,182,183,184,185,186,187,188,189,190,191,192,193,194,195,196,197,198,199,200,201,202,203,204,205,206,207,208,209,210,211,212,213,214,215,216,217,218,219,220,221,222,223,224,225,226,227,228,229,230,231,232,233,234,235,236,237,238,239,240,241,242,243,244,245,246,247,248,249,250,251,252,253,254] (Figure 1).

A detailed summary of all included reviews, including study characteristics and key findings, is provided in Supplementary Table S6.

### 3.2. Methodological Quality Assessment

Among the 250 included systematic reviews, 73 (29.2%) were rated as having high methodological quality, 65 (26.0%) as moderate, 57 (22.8%) as low, and 55 (22.0%) as critically low, based on the AMSTAR 2 tool (Supplementary Table S4). To evaluate the degree of overlap among included reviews, we calculated the CCA for each gastrointestinal surgical domain. The CCA values for esophageal (8.97%), gastric (6.81%), and liver (8.93%) domains indicated moderate overlap. In contrast, the biliary (0.61%), pancreatic (0.02%), and colorectal (0.01%) domains demonstrated slight overlap (Supplementary Table S5). These findings suggest that while redundancy among primary studies was modest in the upper gastrointestinal domains, it was minimal in the lower gastrointestinal and hepatobiliary categories. The quality assessment for each domain is provided in Table 1, and a detailed summary of the general characteristics and key findings of each included article is presented in Supplementary Table S6.

### 3.3. Esophageal Surgery

Robotic esophageal surgery encompasses a wide range of techniques (McKeown, Ivor Lewis, and Heller myotomy), and the included reviews differed substantially in how deeply they analyzed each approach. This variation in procedural focus contributes to heterogeneity in the reported short-term and oncologic outcomes.

#### 3.3.1. Technical Considerations

**Figure 2:**
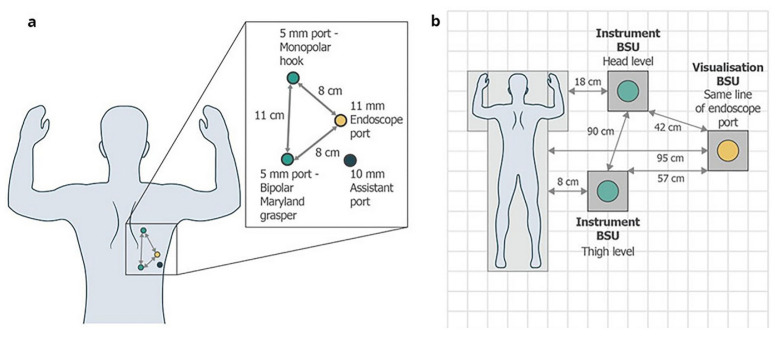
Port positioning and operating room layout. (**a**) Port positioning for TTE and (**b**) corresponding BSU positions. The TTE was a minimal access McKeown’s procedure with cervical esophagogastric anastomosis, performed using a three-hole approach. An 11 mm endoscope port was placed in the 5th or 6th intercostal space. The right 5 mm instrument port was placed approximately in the 3rd intercostal space. The left 5 mm instrument port was placed in the 7th–8th intercostal space. One 10 mm assistant port was placed between the left instrument port and the endoscope port. BSU: bedside unit, TTE: transthoracic esophagectomy. Reproduced from [Puntambekar et al., Sci Rep, 2022 [255]] under the terms of the Creative Commons Attribution 4.0 International License (http://creativecommons.org/licenses/by/4.0/).

Robotic surgery in esophageal procedures offers unique technical advantages over laparoscopic approaches, particularly in the confined mediastinal space. The rigid, straight laparoscopic instruments limit maneuverability during critical steps such as lymphadenectomy near the recurrent laryngeal nerve and thoracic duct dissection. In contrast, robotic systems provide wristed instrumentation and stable 3D visualization, facilitating fine dissection and improved access, especially in the upper mediastinum during esophagectomy [9,10,11]. These benefits are particularly evident in complex procedures such as the McKeown and Ivor Lewis esophagectomies. The McKeown approach (Figure 2) involves three surgical fields—abdominal, thoracic, and cervical—with a cervical anastomosis that allows for extended lymphadenectomy but carries a higher risk of recurrent laryngeal nerve injury [255]. The Ivor Lewis approach, by contrast, is a two-field technique with an intrathoracic anastomosis, offering lower anastomotic stricture rates but posing greater risk if a leak occurs. Robotic platforms enhance precision during these procedures, particularly in esophagogastric anastomosis and lymph node retrieval, contributing to oncologic adequacy [12]. However, robotic esophagectomy often requires redocking during two-field procedures and may involve a steeper learning curve. While robotic stapling and hand-sewn techniques are increasingly used, the lack of tactile feedback remains a technical challenge during high-tension suturing or dissection near vascular structures [9].

**Figure 3:**
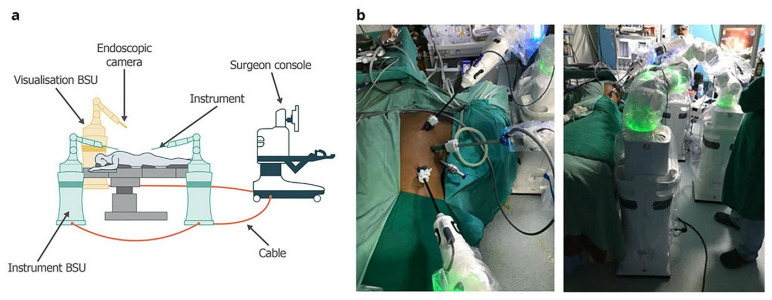
Overview of the Versius Surgical System. Adapted from Haig et al. (**a**) Schematic representation of the setup of Versius and (**b**) real-world images of the Versius setup. BSU: bedside unit. Reproduced from [Puntambekar et al., Sci Rep, 2022 [255]] under the terms of the Creative Commons Attribution 4.0 International License (http://creativecommons.org/licenses/by/4.0/).

**Figure 4:**
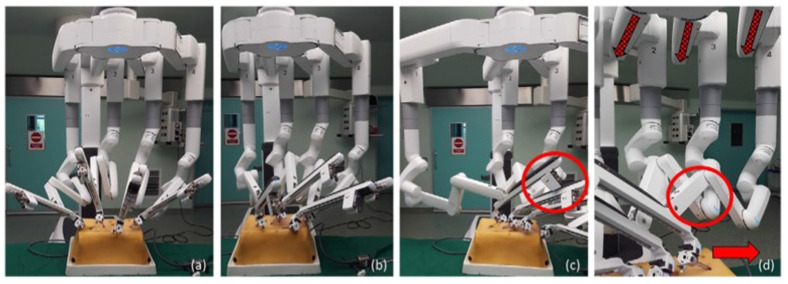
The da Vinci Xi robotic system. (**a**) FLEX joints should be compacted, leaving one-fist-width spacing between each robotic arm (**b**) to allow the robotic arms to move in parallel. (**c**) The instrument carriage tends to clash with the adjacent arm (circle) when the FLEX joints are spaced apart. (**d**) The robotic arms also clash (circle) when the operative target (solid arrow) lies outside of the FLEX joint alignment (dotted arrows). Reproduced from [Ngu et al., RSRR, 2017 [3]] under the terms of the Creative Commons Attribution—NonCommercial (unported, v3.0) License (http://creativecommons.org/licenses/by-nc/3.0/).

Although the Versius system is shown here as an example of emerging robotic platforms (Figure 3), it is important to note that its use in esophageal surgery remains limited. Most of the evidence included in this umbrella review is based on the da Vinci multi-port systems (Figure 4), which currently dominate robotic esophagectomy. The inclusion of Versius reflects the scope of the reviewed literature rather than any direct comparison of platform performance.

#### 3.3.2. Advantages and Limitations

Robotic-assisted minimally invasive esophagectomy (RAMIE) has shown favorable short-term outcomes compared to laparoscopic techniques, including reduced intraoperative blood loss, lower incidence of recurrent laryngeal nerve injury, and fewer pulmonary complications [10,12]. In Heller myotomy for achalasia, the robotic approach is associated with lower rates of mucosal injury and improved postoperative dysphagia scores [5]. Lymph node yield also tends to be higher in RAMIE, reflecting improved precision during mediastinal dissection [13,14]. However, RAMIE is limited by longer operative times, increased setup complexity, and the absence of haptic feedback [9,15]. These challenges can be especially pronounced in patients with obesity or complex mediastinal anatomy. Importantly, long-term oncologic outcomes, including survival and recurrence rates, appear comparable between robotic and laparoscopic approaches [13,16].

#### 3.3.3. Cost-Effectiveness

Despite improved perioperative outcomes, robotic esophagectomy remains more costly than laparoscopic approaches due to the high capital expense of robotic systems, longer operative times, and specialized instrumentation [17,18]. However, in complex cases such as redo surgery or paraesophageal hernia repair, robotic assistance may reduce healthcare costs associated with postoperative complications and overall hospitalization, thereby partially offsetting the initial investment [19,20]. Therefore, while RAMIE may not be cost-effective for routine cases, its value increases in technically demanding scenarios.

### 3.4. Gastric Surgery

Gastric surgery represents one of the most heterogeneous domains in robotic GI surgery, with differences in distal versus total gastrectomy, D2 lymphadenectomy, and reconstruction techniques. The included reviews vary considerably in the depth of analysis across these subprocedures, which explains the diversity in reported outcomes.

#### 3.4.1. Technical Considerations

**Figure 5:**
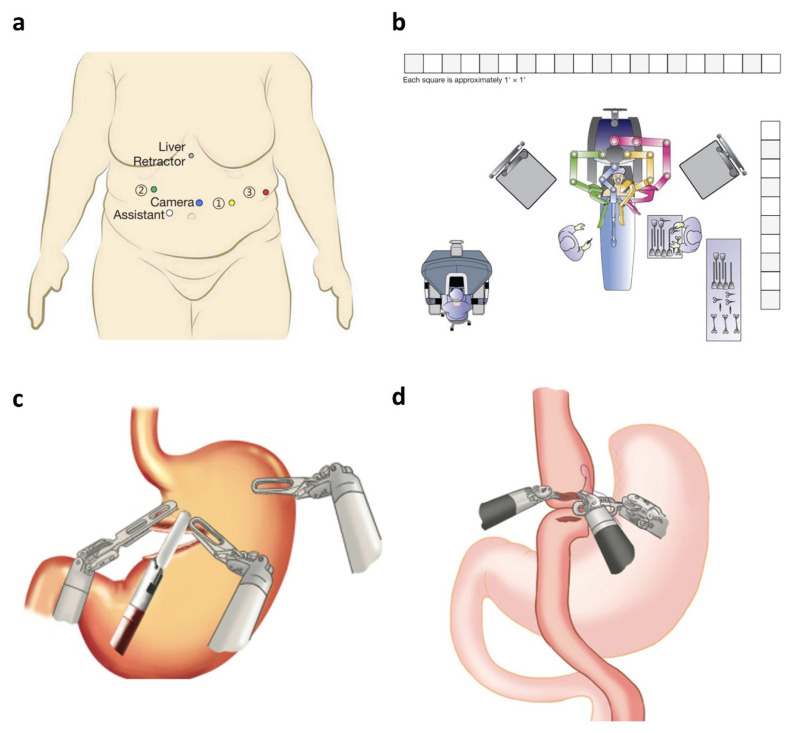
Robotic Roux-en-Y gastric bypass. (**a**) Port position in robotic gastric bypass. (**b**) Operating room setup and patient cart positioning for robot-assisted Roux-en-Y gastric bypass (RYGB). (**c**) Sketch diagram showing horizontal stapler fire for formation of gastric pouch. (**d**) Sketch diagram showing creation of the gastrojejunostomy (GJ). A hand-sewn GJ is being created. The third arm is holding the gastric pouch and Roux limb together. Reproduced from [Bindal et al., Dig Med Res, 2021 [256]] under the terms of the Creative Commons Attribution—NonCommercial—NoDerivatives 4.0 International License (https://creativecommons.org/licenses/by-nc-nd/4.0/).

Gastric cancer surgery often requires extensive lymphadenectomy and precise anastomosis within a confined space. While laparoscopic gastrectomy has been widely adopted, it remains technically challenging for D2 lymph node dissection and intracorporeal reconstruction [33]. Robotic gastrectomy provides greater articulation through wristed instruments, allowing for improved maneuverability around major vessels such as the left gastric artery and splenic hilum [34,35,36]. Additionally, the robotic platform facilitates suturing during intracorporeal Billroth I/II or Roux-en-Y reconstruction (Figure 5), especially in high BMI patients or those with visceral obesity [37,38,39,256]. The stable camera platform and 3D visualization enhance the precision of perigastric dissection, contributing to better lymph node yield and fewer vascular injuries [40]. However, robotic systems also introduce limitations such as increased setup time, limited haptic feedback, and instrument clashing in narrow pelvic anatomy [39,40,41].

#### 3.4.2. Advantages and Limitations

Robotic gastrectomy (RG) is associated with significantly reduced intraoperative blood loss, lower conversion rates, and fewer postoperative complications compared to laparoscopic gastrectomy (LG) [42,43]. These advantages are most apparent in technically demanding situations, particularly in cases requiring D2 lymphadenectomy, where robotic assistance enables more precise dissection and yields higher lymph node retrieval without increasing operative morbidity [36,44]. The oncologic importance of achieving an adequate D2 lymphadenectomy has been repeatedly emphasized, and several studies suggest that robotic systems may facilitate more consistent nodal dissection due to improved visualization and instrument dexterity [45]. RG has also demonstrated reduced pancreatic fistula rates in overweight patients and may offer additional functional benefits, such as improved gastric conduit preservation [46].

Despite these advantages, RG is associated with longer operative times and a prolonged learning curve, particularly for total gastrectomy. Furthermore, several studies report no significant difference in long-term oncologic outcomes—such as recurrence or overall survival—between RG and LG [40,47].

#### 3.4.3. Cost-Effectiveness

Robotic gastrectomy entails higher upfront costs related to robotic system acquisition, maintenance, and disposable instrumentation. Studies have shown that RG increases operative cost compared to LG by approximately 1.3–1.7 times [41,48,49]. However, this may be partially offset by fewer complications, lower readmission rates, and shorter recovery time in high-risk or obese patients [33,50]. Despite these potential benefits, cost-effectiveness remains limited in standard-risk patients undergoing routine gastrectomy.

### 3.5. Liver Surgery

Robotic liver resection covers a broad spectrum of procedures, from minor hepatectomy to major posterosuperior segmentectomy and bile duct reconstruction. The included reviews reflect this procedural diversity, leading to variability in the emphasis placed on technical complexity and perioperative outcomes.

#### 3.5.1. Technical Considerations

**Figure 6:**
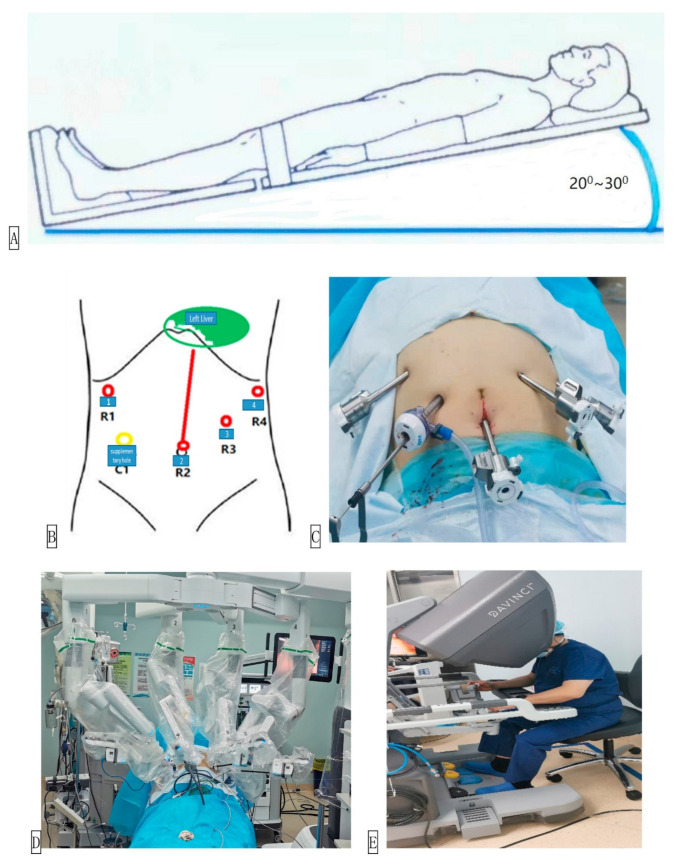
The operation layout of robot assisted hepatectomy. (**A**) Position of the patient. (**B**) Position of the operating hole. (**C**) Photo of the operating hole. (**D**) Da Vinci Xi™ robot (Intuitive Surgical, Sunnyvale, CA, USA) and assistant during operation. (**E**) The surgeon controls the Da Vinci Xi™ robot on the surgeon’s console. Reproduced from [Sun et al., Intelligent Surgery, 2022 [257]] under the terms of the Creative Commons Attribution—NonCommercial—NoDerivatives 4.0 International License (https://creativecommons.org/licenses/by-nc-nd/4.0/).

**Figure 7:**
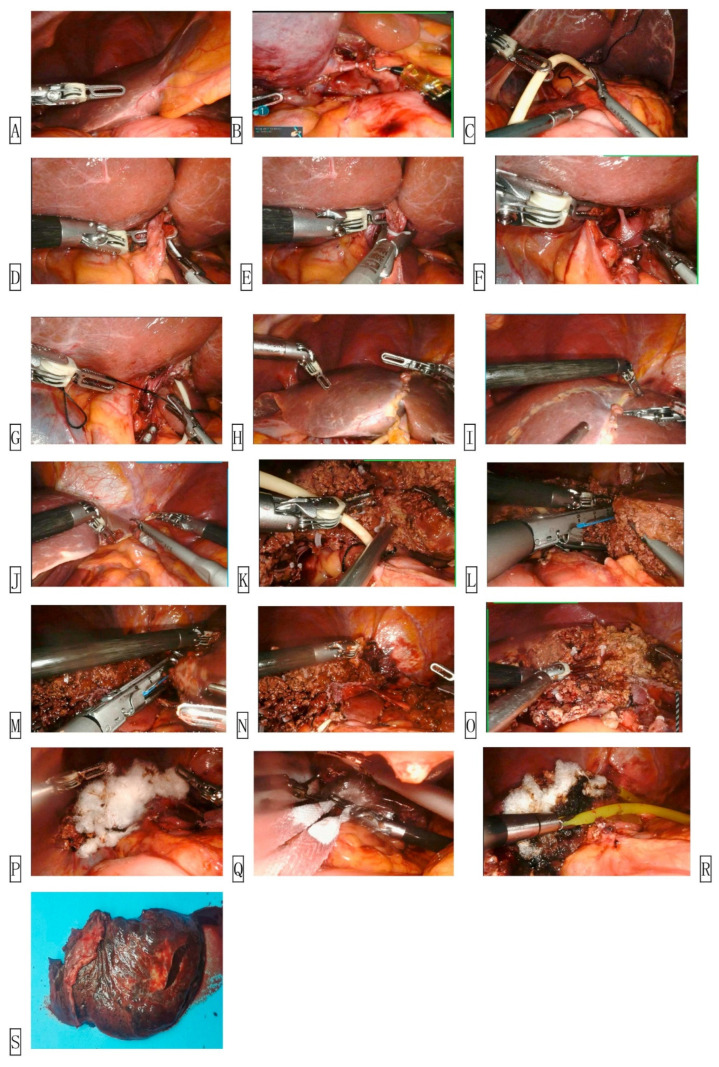
Operation Steps of Robot Assisted Hepatectomy. (**A**) Explore the abdominal cavity. (**B**) Cholecystectomy. (**C**) Place hepatic port blocking tape. (**D**) Isolate the left hepatic artery. (**E**) Ligate the left hepatic artery. (**F**) Isolate the left portal vein. (**G**) Ligate the left portal vein. (**H**) View the ischemic line. (**I**) Line the ischemia line. (**J**) Detach the left deltoid ligament. (**K**) Severed liver. (**L**) Detach the left liver pedicle. (**M**,**N**) Cut off the left hepatic vein. (**O**) Hepatic hemostasis. (**P**) Place hemostatic yarn. (**Q**) Remove the specimen. (**R**) Place drainage tube. (**S**) Sample of left liver. Reproduced from [Sun et al., Intelligent Surgery, 2022 [257]] under the terms of the Creative Commons Attribution—NonCommercial—NoDerivatives 4.0 International License (https://creativecommons.org/licenses/by-nc-nd/4.0/).

Liver resection presents specific challenges related to vascular control, parenchymal transection, and access to posterosuperior segments. Laparoscopic liver surgery often relies on the Cavitron Ultrasonic Surgical Aspirator (CUSA) for precise parenchymal dissection, which cannot be directly controlled by the robotic console. Instead, robotic systems utilize alternative tools such as bipolar forceps or vessel sealers, often requiring bedside assistant coordination [77,78,79]. The robotic approach, however, provides enhanced access to difficult segments (VII and VIII) due to wristed instruments and improved ergonomics [80,81,82]. Robotic visualization also facilitates fine suturing in bile duct reconstructions and hepaticojejunostomies, although the lack of haptic feedback may pose risks during deep dissection or vascular clipping [77,83,84,85]. Moreover, robotic resections (Figure 6 and Figure 7) require careful port placement and repositioning when switching between hepatic lobes [257].

Intraoperative ultrasound also plays a crucial role in robotic liver surgery, enabling accurate demarcation of tumor margins and helping guide safe parenchymal transection at an appropriate distance from the lesion.

#### 3.5.2. Advantages and Limitations

Robotic liver resection (RLR) is associated with reduced blood loss, lower conversion rates, and shorter hospital stays compared to open or laparoscopic approaches in major hepatectomies [79,80,86]. Its advantages are especially pronounced in complex resections or in patients with cirrhosis, where reduced bleeding and improved access may reduce postoperative morbidity [87]. Studies also report comparable oncologic outcomes for hepatocellular carcinoma and colorectal liver metastasis between robotic and laparoscopic techniques [88,89]. However, RLR involves significantly longer operative times, and its clinical efficacy remains debated in the literature, with some studies reporting outcomes comparable to conventional approaches without demonstrating clear superiority [90,91,92]. Additionally, the absence of tactile sensation and high dependency on the bedside assistant for tasks like suctioning or CUSA operation can limit intraoperative autonomy [77,87,93].

#### 3.5.3. Cost-Effectiveness

Robotic liver resection incurs higher direct costs due to robotic instruments, maintenance fees, and increased operating room time. Nonetheless, economic analyses indicate that RLR may be cost-effective in selected high-complexity cases by reducing conversion and complication rates [94,95]. In patients requiring resections of posterosuperior segments or bile duct reconstructions, robotic surgery may offer cost-offsetting benefits by minimizing ICU duration and readmissions. In routine minor resections, however, laparoscopic techniques remain more cost-efficient [89,96].

### 3.6. Biliary Surgery

Robotic biliary surgery includes both routine benign procedures and highly complex oncologic resections, such as hilar cholangiocarcinoma. Because the included reviews focus on different procedural subsets, the depth and scope of analysis vary accordingly across studies.

#### 3.6.1. Technical Considerations

**Figure 8:**
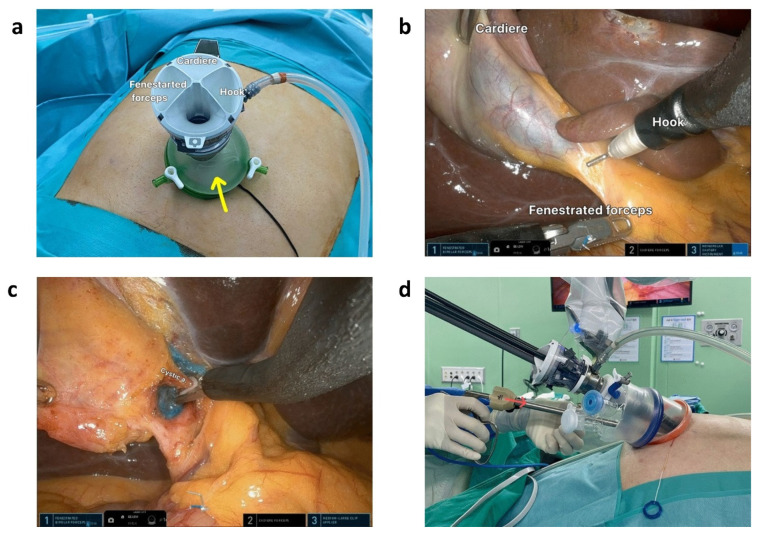
Single-incision robotic cholecystectomy using the da Vinci Single-Port (SP) robotic surgical system. (**a**) The SP system has three robotic arms that can be controlled by the operator. (**b**) The middle arm, in this case, the Cardiere forceps arm, is used for gallbladder traction. (**c**) The multidirectional Endo-Wrist allows approaching the surgical field with the appropriate angle. (**d**) In acute cholecystitis, the cystic duct may become dilated, making it difficult to ligate using a typical single-size medium-large (green) robotic hemolock. In the SP system, the assistant can insert a larger (purple size; red arrow) hemolock through the umbilical port to clip the cystic duct. Reproduced from [Choi et al., Sci Rep, 2023 [258]] under the terms of the Creative Commons Attribution 4.0 International License (https://creativecommons.org/licenses/by/4.0/).

Surgical robots feature wristed instruments with increased flexibility and enhanced freedom of movement (Figure 8), offering technical advantages in complex cases and patients with altered foregut anatomy [110,111,112]. In demanding procedures, such as resections for hilar cholangiocarcinoma, robotic systems facilitate meticulous dissection of critical structures including the hepatic artery, portal vein, and biliary confluence within a confined space [113,114]. Lymphadenectomy around the hepatoduodenal ligament is also enhanced by the tremor filtration and stable camera platform of robotic system, supporting precise nodal dissection without vascular injury [110,115]. Robotic platforms improve intracorporeal suturing during hepaticojejunostomy, enabling secure bilioenteric anastomosis with reduced tension and better visualization [115,116,117]. This precision is particularly beneficial in patients with inflammatory changes, advanced liver disease, or elevated BMI [118,258].

#### 3.6.2. Advantages and Limitations

Robotic biliary surgery demonstrates comparable short-term outcomes to laparoscopic surgery in benign disease, with similar complication rates, blood loss, and hospital stay [111,119,120]. With ongoing technological advancements and growing surgical expertise, its application has expanded to more complex procedures, including resections for hilar cholangiocarcinoma [116,121,122,123]. However, operative time is generally longer for robotic procedures, particularly in the learning phase [119,120]. Long-term oncologic outcomes, including survival and recurrence rates, are comparable between robotic and laparoscopic approaches for gallbladder cancer and cholangiocarcinoma [115,116]. Limitations of robotic biliary surgery include the lack of haptic feedback, steep learning curve for complex reconstructions, and restricted access in emergency settings or low-resource environments [123].

#### 3.6.3. Cost-Effectiveness

Robotic biliary surgery incurs higher direct costs than laparoscopic surgery, largely due to robotic system acquisition, maintenance, and instrument expenses [124]. Increased operative time and disposable costs further contribute to the financial burden [119,120,121]. While shorter hospital stays and reduced conversion rates may partially offset expenses in complex cases, routine robotic cholecystectomy has not demonstrated cost-effectiveness over laparoscopy in benign disease [120]. Robotic surgery may provide greater economic value in high-risk or technically demanding cases, such as hilar cholangiocarcinoma, where improved surgical precision may reduce postoperative complications and long-term morbidity [123].

### 3.7. Pancreatic Surgery

Robotic pancreatic surgery encompasses technically distinct procedures, including distal pancreatectomy and pancreaticoduodenectomy, which differ markedly in complexity. The included reviews emphasize these subprocedures to varying degrees, contributing to heterogeneity in the range and depth of reported outcomes.

#### 3.7.1. Technical Considerations

**Figure 9:**
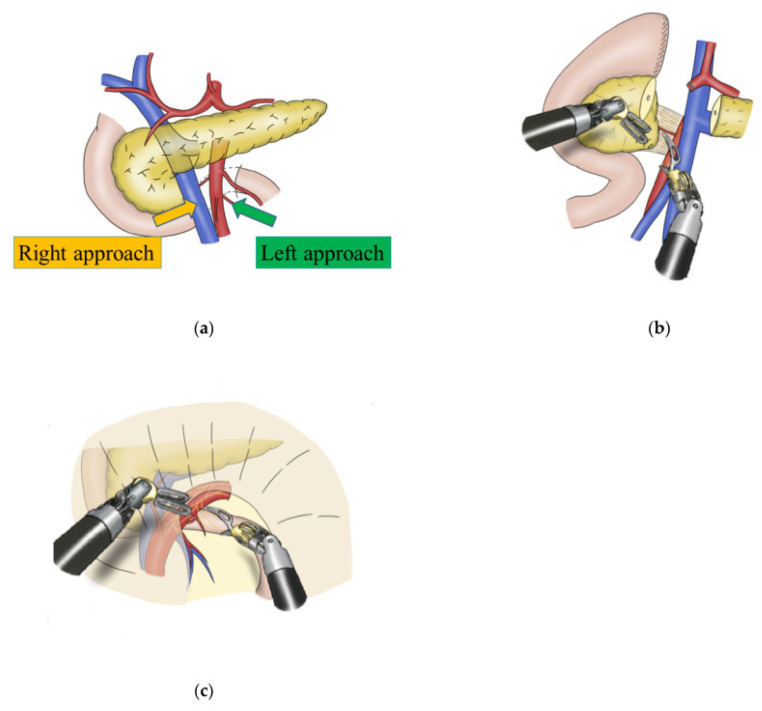
Surgical approaches to the superior mesenteric artery (SMA) in robotic pancreaticoduodenectomy. (**a**) Schematic view of the right and left approaches to the SMA. (**b**) The right approach is considered as the standard protocol for RPD. (**c**) The left approach is used in patients with obesity, intra-abdominal adhesions, or malignant diseases requiring lymph node dissection around the SMA. Reproduced from [Takagi et al., JCM, 2022 [259]] under the terms of the Creative Commons Attribution 4.0 International License (https://creativecommons.org/licenses/by/4.0/).

Pancreatic surgery poses unique technical challenges due to the retroperitoneal location, proximity to major vascular structures, and the complexity of anastomotic reconstruction. In robotic distal pancreatectomy (DP), the wristed instruments facilitate precise dissection along the splenic artery and enable delicate mobilization of the pancreas, especially in spleen-preserving approaches [140]. However, parenchymal transection remains reliant on laparoscopic energy devices or robotic staplers, as robotic-compatible CUSA systems are not widely available [141]. In robotic pancreaticoduodenectomy (PD), stable 3D visualization supports accurate vascular control during dissection of the superior mesenteric vessels (Figure 9), and enhanced dexterity aids in performing duct-to-mucosa pancreaticojejunostomy with finer suturing [142,143,144,259]. Lymphadenectomy around the hepatoduodenal ligament and interaortocaval regions is also facilitated by the robot’s precision in confined retroperitoneal planes. Nevertheless, the complexity of robotic PD demands significant operative experience and institutional infrastructure, particularly for safe execution of anastomoses and vascular management. Limited haptic feedback may affect depth perception during critical steps, requiring reliance on visual cues and surgeon expertise [145].

#### 3.7.2. Advantages and Limitations

In distal pancreatectomy (DP), robotic surgery has demonstrated favorable short-term outcomes compared to the laparoscopic approach, including lower conversion rates, reduced intraoperative blood loss, and higher spleen preservation rates, particularly in challenging dissections near the splenic hilum [140]. In pancreaticoduodenectomy (PD), robotic surgery achieves similar morbidity and mortality rates compared to laparoscopic and open approaches, with improved anastomotic precision and potential reductions in delayed gastric emptying and wound infection [146]. Oncologic outcomes, such as R0 resection rates and lymph node yields, are comparable across techniques [147]. However, limitations of robotic pancreatic surgery include prolonged operative times, especially during the learning curve, and the lack of tactile sensation during vascular dissection. Additionally, robotic PD remains restricted to high-volume centers due to its technical demands and resource intensity [145]. Long-term functional outcomes are not yet well defined in the current literature and warrant further study. Access and surgeon training also represent barriers to wider adoption, particularly in low-resource or non-academic centers.

Despite the technical advantages of robotic assistance, postoperative pancreatic fistula remains a significant risk following both distal pancreatectomy and pancreaticoduodenectomy, underscoring the intrinsic complexity of pancreatic surgery regardless of platform.

#### 3.7.3. Cost-Effectiveness

Robotic pancreatic surgery generally incurs higher overall costs compared to laparoscopic and open approaches. The primary cost drivers include robotic system acquisition, maintenance, and the use of disposable instruments [148]. Longer operative times further contribute to increased operating room costs. However, in the case of robotic DP, procedural costs may decrease with surgical experience. Studies suggest that after a minimum of five cases, operative efficiency improves and costs begin to decline, highlighting the importance of structured training [149]. Furthermore, robotic DP and PD have demonstrated advantages including shorter hospital stays, lower conversion rates, and fewer complications, which may contribute to cost mitigation [148,149,150]. Nonetheless, these benefits have not consistently translated into overall cost savings across institutions. Thus, while the economic justification for robotic surgery remains weak in routine cases, its application may be more favorable in complex or high-risk patients, where its technical advantages can reduce perioperative morbidity and reoperation risk.

### 3.8. Colorectal Surgery

Robotic colorectal surgery spans procedures of varying difficulty, from right colectomy to deep pelvic total mesorectal excision and IPAA. The included reviews focus on different colorectal subsites, which explains the variability in the level of detail across outcome measures.

#### 3.8.1. Technical Considerations

**Figure 10:**
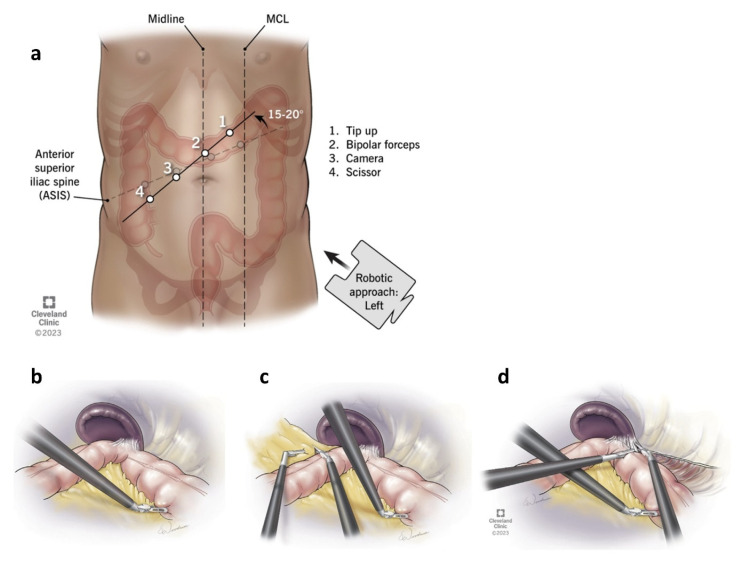
Splenic flexure mobilization in rectal surgery, using the da Vinci Xi robotic system in a cross-armed single-docking approach. First, a tip-up fenestrated grasper inserted through the port number one retracts the descending colon. Then, the arms are crossed either from the medial or lateral aspect of arm one to take down the flexure. (**a**) The standard port placement line was moved 15–20° counterclockwise to facilitate mobilization. (**b**) Traction of descending colon. (**c**) Crossover from the medial aspect of arm one. (**d**) Crossover from the lateral aspect of arm one. Reproduced from [Erozkan et al., MIS, 2023 [260]] under the terms of the Creative Commons Attribution 4.0 International License (https://creativecommons.org/licenses/by/4.0/).

Robotic colorectal surgery provides distinct technical advantages in procedures involving complex pelvic dissection and challenging anastomoses. In total mesorectal excision (TME) for rectal cancer, the robotic system enhances precision during sharp dissection within the narrow pelvis, facilitating preservation of the hypogastric nerves and pelvic autonomic plexus [178,179]. Pelvic lateral lymph node dissection, which is technically challenging laparoscopically, may also be facilitated by robotic precision in advanced rectal cancer cases [180]. Robotic articulation also enables stable and accurate dissection along the mesorectal plane, reducing the risk of circumferential resection margin involvement [181]. In ileal pouch-anal anastomosis (IPAA), the platform improves suturing in deep pelvic spaces, contributing to secure anastomosis and sphincter preservation [182]. Robotic right colectomy benefits from intracorporeal anastomosis, allowing improved vascular control and tension-free anastomotic construction [183]. Although CUSA is rarely used in colorectal surgery, energy devices such as vessel sealers and robotic staplers play a critical role in mesenteric dissection and division. However, robotic dissection in obese patients or those with bulky tumors remains technically demanding despite improved access angles [184,185,186]. Splenic flexure mobilization is another challenging step in rectal surgery, but many technical barriers have been mitigated with surgical robot evolution (Figure 10). For instance, the da Vinci Xi system offers a clear advantage over its predecessor, the da Vinci Si, by enabling multi-quadrant surgery without the need for redocking. This allows for a more efficient single-docking approach, reducing operative time and improving access for procedures such as rectosigmoid resection and splenic flexure takedown [260]. Thus, colorectal surgery stands to benefit further from innovations that enhance precision, efficiency, and operative flexibility across the abdominal quadrants.

#### 3.8.2. Advantages and Limitations

Robotic colorectal surgery demonstrates improved short-term outcomes compared to laparoscopy in terms of overall complication rates, blood loss, and hospital stay [187,188]. Especially, robotic rectal resection is associated with significantly lower conversion rates to open surgery and improved quality of TME, particularly in male patients with a narrow pelvis or obese patients [189]. In selected cases, robotic surgery enables higher lymph node harvest and improved preservation of urinary and sexual function, attributed to superior nerve-sparing dissection [190]. Long-term oncologic outcomes, including disease-free survival and local recurrence rates, are generally comparable between robotic, laparoscopic, and open approaches [191,192]. Limitations of robotic colorectal surgery include prolonged operative time, particularly during the learning phase, lack of tactile feedback, and high dependence on institutional resources and surgeon expertise [193]. In addition, robotic appendectomy and routine colectomy for benign disease have not demonstrated clear superiority over laparoscopic techniques in uncomplicated cases [194].

#### 3.8.3. Cost-Effectiveness

Robotic colorectal surgery is consistently associated with higher direct costs than laparoscopic approaches due to equipment acquisition, maintenance fees, and use of proprietary instruments [195]. Increased operative time further adds to the total surgical cost. Although the cost-effectiveness of robotic colectomy for benign disease remains limited, reduced conversion rates, shorter hospital stays, and improved functional outcomes may offset expenses in complex pelvic procedures [196]. Current evidence suggests that robotic surgery may provide greater economic value in high-risk or technically demanding cases, such as low rectal cancer or IPAA, where its technical advantages can lead to improved perioperative outcomes and reduced long-term morbidity [182,197].

## 4. Discussion

Robotic surgery has advanced gastrointestinal (GI) surgery by offering enhanced visualization, dexterity, and instrument control in anatomically complex spaces. However, its clinical value is not uniform across surgical domains and depends heavily on procedural complexity, institutional volume, and resource availability. A cross-domain comparative summary of robotic GI surgery is provided in Table 2.

Among the six domains, robotic assistance appears to confer the most consistent clinical advantage in pancreatic and colorectal surgery. Clinically, robotic assistance enhances dissection in dense anatomical planes, reduces conversion rates, and shortens hospital stays. These benefits are particularly evident in demanding procedures such as total mesorectal excision and pancreaticoduodenectomy, where the precision of robotic systems improves safety and technical outcomes. Economically, both procedures may achieve cost-efficiency in high-volume centers by reducing complications and facilitating faster recovery.

Despite these advantages, certain procedures with strong theoretical benefits remain underutilized in robotic practice. For example, robotic liver surgery offers enhanced access and fine control, particularly in posterior segment resections, yet its adoption remains limited due to technical demands, steep learning curves, and a lack of high-quality data. This mismatch between potential benefit and real-world adoption warrants attention. Structured training programs and multi-institutional collaborations could help standardize technique and generate stronger evidence to guide implementation.

Moving forward, the expansion of robotic surgery should be driven by clinical value, not technological novelty. While robotic systems provide significant advantages in certain GI procedures, their optimal use depends on thoughtful procedural selection, institutional expertise, and ongoing evidence generation. Integration of robotic platforms must be guided by procedure-specific outcomes, long-term oncologic safety, and cost-effectiveness. Future research should prioritize comparative studies across diverse healthcare settings, especially in lower-volume centers and regions with constrained surgical access, to evaluate scalability and equity in robotic care delivery. Addressing the gap between potential and practice will be essential in shaping the next phase of robotic gastrointestinal surgery.

This umbrella review has several inherent limitations. First, the analysis depends entirely on previously published systematic reviews and meta-analyses, and therefore inherits their methodological variability, including differences in search scope, patient selection, and outcome definitions. Second, comparator heterogeneity—some reviews comparing robotic surgery to laparoscopic approaches, others to open surgery—limits the ability to standardize effect estimates across domains. Third, variations in robotic platforms across studies (e.g., da Vinci, SP, Versius, Senhance) introduce additional heterogeneity, as most evidence remains dominated by da Vinci systems. Finally, because umbrella reviews do not reanalyze primary data, the depth of procedure-specific comparisons is constrained by the granularity of the included reviews. These limitations should be considered when interpreting the findings.

## 5. Conclusions

This umbrella review analyzed 250 studies across six GI surgical domains to assess the comparative value of robotic surgery. Robotic platforms enhance dexterity, visualization, and ergonomics, with the most notable benefits being seen in complex pancreatic and colorectal procedures. These cases show lower conversion rates and fewer complications, with outcomes that are comparable to conventional approaches. While high costs remain a challenge, the findings of this review may serve as a valuable resource for clinicians, surgical trainees, and healthcare decision-makers evaluating the appropriate integration of robotic platforms into gastrointestinal surgical practice. Continued technological refinement, coupled with rigorous clinical research, will be essential to defining the optimal role of robotic surgery.

## Figures and Tables

**Table 1:** Quality assessment of the studies included in each surgical domain.

Domain	Number of Reviews	AMSTAR 2 Evaluation	CCA Value (%)	Overlap Level
Esophageal	25	High: 10, Moderate: 0, Low: 4, Critically low: 11	8.97%	Moderate
Gastric	44	High: 0, Moderate: 30, Low: 10, Critically low: 4	6.81%	Moderate
Liver	33	High: 16, Moderate: 11, Low: 1, Critically low: 5	8.93%	Moderate
Biliary	30	High: 5, Moderate: 0, Low: 9, Critically low: 16	0.61%	Slight
Pancreatic	40	High: 15, Moderate: 2, Low: 14, Critically low: 9	0.02%	Slight
Colorectal	78	High: 27, Moderate: 22, Low: 19, Critically low: 10	0.01%	Slight

AMSTAR 2: A Measurement Tool to Assess Systematic Reviews 2; CCA: Corrected Covered Area.

**Table 2:** Cross-domain comparative summary of robotic gastrointestinal surgery.

Domain	Optimal Indications	Established Clinical Benefits	Cost-Effectiveness	Evidence-Based Conclusion
Esophageal	McKeown or Ivor Lewis esophagectomy; Heller myotomy	Lower pulmonary and nerve injury rates; Improved lymph node yield	Potentially justified in complex cases	Selectively recommended for complex cases.
Gastric	D2 lymphadenectomy; High-risk or obese patients	Lower conversion and complication rates; Improved lymphadenectomy and anastomotic precision	Moderate only in high-risk cases	Selectively recommended for high-risk cases; cost restricts broad adoption.
Liver	Posterior segmentectomy; Bile duct reconstruction; Cirrhotic liver	Lower conversion rates; Shorter hospital stays; Enhanced access in complex resections	Favorable only in complex resections	Selectively recommended for complex cases; cost and debated clinical superiority restrict routine use.
Biliary	Hilar cholangiocarcinoma; Hepaticojejunostomy; High BMI or inflammation	Enhanced dissection and suturing in confined spaces; Improved lymphadenectomy and anastomotic precision; Comparable in benign disease	Potentially justified in complex or high-risk oncologic cases	Selectively recommended for high-risk cases; further evidence needed to justify broader adoption.
Pancreatic	PD; Spleen-preserving DP; High-risk or obese patients	Lower conversion rates; Improved anastomotic precision in PD; Comparable oncologic outcomes	Favorable in complex cases and high-volume centers; Improves with training and experience	Strongest evidence of clinical value in high-risk cases or advanced centers; not yet scalable for routine implementation.
Colorectal	TME; IPAA; Rectal cancer in male or obese patients	Lower complication and conversion rates; Shorter hospital stays; Improved nerve preservation	Reasonable in complex cases; Justified by reduced morbidity and enhanced functional outcomes	Substantial advantages in high-risk pelvic cases, particularly rectal cancer; cost restricts routine use for benign conditions.

BMI: body mass index; PD: pancreaticoduodenectomy; DP: distal pancreatectomy; TME: total mesorectal excision; IPAA: ileal pouch-anal anastomosis.

## Data Availability

This study is an umbrella review and does not involve the collection or generation of original data. All data analyzed were derived from previously published studies, which are publicly available through the original publications. The data supporting the findings of this study are available from the corresponding author upon reasonable request.

## Supplementary Materials

The following supporting information can be downloaded at https://www.mdpi.com/article/10.3390/jcm14238555/s1: Table S1: Full Search Strategy; Table S2: PRISMA 2020 Checklist; Table S3: PRISMA Abstract Checklist; Table S4: AMSTAR 2 evaluation of the key studies in each surgical domain; Table S5: CCA calculation using a citation matrix of eligible reviews in each surgical domain; Table S6: General characteristics of the 250 articles included in this umbrella review.

## Abbreviations

The following abbreviations are used in this manuscript:

AMSTAR2	A Measurement Tool to Assess Systematic Reviews 2
BMI	Body Mass Index
CCA	Corrected Covered Area
CUSA	Cavitron Ultrasonic Surgical Aspirator
DP	Distal Pancreatectomy
GI	Gastrointestinal
IPAA	Ileal Pouch-Anal Anastomosis
LG	Laparoscopic Gastrectomy
MIS	Minimally Invasive Surgery
PD	Pancreaticoduodenectomy
RAMIE	Robotic-Assisted Minimally Invasive Esophagectomy
RAS	Robotic-Assisted Surgery
RG	Robotic Gastrectomy
RLR	Robotic Liver Resection
SP	Single-Port
TME	Total Mesorectal Excision
