# Supplementary material for: Robotic Gastrointestinal Surgery Compared to Conventional Approaches: An Umbrella Review of Clinical and Economic Outcomes
Source: J Clin Med. 2025 Dec 2;14(23):8555. doi: 10.3390/jcm14238555 (PMC12693434; doi:10.3390/jcm14238555)
Supplement: Supplementary file 1 [file jcm-14-08555-s001.zip › jcm-3962703-supplementary.pdf]

Table S1. Full Search Strategy.

Domain	Search Term
Esophageal	(("robot" OR "robotic" OR "da Vinci Surgical System") AND ("laparoscopic" OR "open" OR "conventional") AND ("esophagus" OR "esophageal" OR "oesophagus" OR "oesophageal" OR "esophagectomy" OR "oesophagectomy" OR "fundoplication" OR "hiatal" OR "reflux" OR "diverticulectomy" OR "esophagomyotomy") AND ("operative" OR "surgical" OR "outcome" OR "cost"))
Gastric	(("robot" OR "robotic" OR "da Vinci Surgical System") AND ("laparoscopic" OR "open" OR "conventional") AND ("stomach" OR "gastric" OR "gastrectomy" OR "bariatric" OR "gastric bypass" OR "sleeve gastrectomy") AND ("operative" OR "surgical" OR "outcome" OR "cost"))
Liver	(("robot" OR "robotic" OR "da Vinci Surgical System") AND ("laparoscopic" OR "open" OR "conventional") AND ("liver" OR "hepatic" OR "hepatectomy" OR "liver resection" OR "liver transplant" OR "liver ablation") AND ("operative" OR "surgical" OR "outcome" OR "cost"))
Biliary	(("robot" OR "robotic" OR "da Vinci Surgical System") AND ("laparoscopic" OR "open" OR "conventional") AND ("biliary" OR "bile duct" OR "cholecystectomy" OR "gallbladder") AND ("operative" OR "surgical" OR "outcome" OR "cost"))
Pancreatic	(("robot" OR "robotic" OR "da Vinci Surgical System") AND ("laparoscopic" OR "open" OR "conventional") AND ("pancreas" OR "pancreatic" OR "pancreatectomy" OR "pancreaticoduodenectomy" OR "Whipple procedure") AND ("operative" OR "surgical" OR "outcome" OR "cost"))
Colorectal	(("robot" OR "robotic" OR "da Vinci Surgical System") AND ("laparoscopic" OR "open" OR "conventional") AND ("colorectal" OR "colectomy" OR "colostomy" OR "polypectomy" OR "proctectomy" OR "strictureplasty" OR "ileostomy" OR "colorectal resection" OR "rectal" OR "rectum" OR "appendix" OR "appendectomy" OR "appendicectomy") AND ("operative" OR "surgical" OR "outcome" OR "cost"))

Table S2. PRISMA 2020 Checklist.

Section and Topic	Item #	Checklist item	Location where item is reported
**TITLE**			
Title	1	Identify the report as a systematic review.	Title page
**ABSTRACT**			
Abstract	2	See the PRISMA 2020 for Abstracts checklist.	All checklist items are reported
**INTRODUCTION**			
Rationale	3	Describe the rationale for the review in the context of existing knowledge.	Introduction (Page 4–5)
Objectives	4	Provide an explicit statement of the objective(s) or question(s) the review addresses.	Introduction (Page 5)
**METHODS**			
Eligibility criteria	5	Specify the inclusion and exclusion criteria for the review and how studies were grouped for the syntheses.	Methods > Eligibility Criteria
Information sources	6	Specify all databases, registers, websites, organisations, reference lists and other sources searched or consulted to identify studies. Specify the date when each source was last searched or consulted.	Methods > Search Strategy
Search strategy	7	Present the full search strategies for all databases, registers and websites, including any filters and limits used.	Table S1
Selection process	8	Specify the methods used to decide whether a study met the inclusion criteria of the review, including how many reviewers screened each record and each report retrieved, whether they worked independently, and if applicable, details of automation tools used in the process.	Methods > Screening and Data Extraction
Data collection process	9	Specify the methods used to collect data from reports, including how many reviewers collected data from each report, whether they worked independently, any processes for obtaining or confirming data from study investigators, and if applicable, details of automation tools used in the process.	Methods > Screening and Data Extraction

Section and Topic	Item #	Checklist item	Location where item is reported
Data items	10a	List and define all outcomes for which data were sought. Specify whether all results that were compatible with each outcome domain in each study were sought (e.g. for all measures, time points, analyses), and if not, the methods used to decide which results to collect.	Methods > Screening and Data Extraction / Table S5
	10b	List and define all other variables for which data were sought (e.g. participant and intervention characteristics, funding sources). Describe any assumptions made about any missing or unclear information.	Table S6
Study risk of bias assessment	11	Specify the methods used to assess risk of bias in the included studies, including details of the tool(s) used, how many reviewers assessed each study and whether they worked independently, and if applicable, details of automation tools used in the process.	Methods > Methodological Quality Assessment
Effect measures	12	Specify for each outcome the effect measure(s) (e.g. risk ratio, mean difference) used in the synthesis or presentation of results.	N/A – No meta-analysis or effect size synthesis was conducted.
Synthesis methods	13a	Describe the processes used to decide which studies were eligible for each synthesis (e.g. tabulating the study intervention characteristics and comparing against the planned groups for each synthesis (item #5)).	Methods > Surgical domains and grouping described
	13b	Describe any methods required to prepare the data for presentation or synthesis, such as handling of missing summary statistics, or data conversions.	Methods > Screening and Data Extraction
	13c	Describe any methods used to tabulate or visually display results of individual studies and syntheses.	Table 1-2, S4-6<br>Figure 3
	13d	Describe any methods used to synthesize results and provide a rationale for the choice(s). If meta-analysis was performed, describe the model(s), method(s) to identify the presence and extent of statistical heterogeneity, and software package(s) used.	No meta-analysis was done; results were synthesized narratively by surgical domain
	13e	Describe any methods used to explore possible causes of heterogeneity among study results (e.g. subgroup analysis, meta-regression).	Not applicable
	13f	Describe any sensitivity analyses conducted to assess robustness of the synthesized results.	Not conducted

Section and Topic	Item #	Checklist item	Location where item is reported
Reporting bias assessment	14	Describe any methods used to assess risk of bias due to missing results in a synthesis (arising from reporting biases).	Not reported
Certainty assessment	15	Describe any methods used to assess certainty (or confidence) in the body of evidence for an outcome.	Not reported
**RESULTS**			
Study selection	16a	Describe the results of the search and selection process, from the number of records identified in the search to the number of studies included in the review, ideally using a flow diagram.	Results > Study Selection / Figure 3
	16b	Cite studies that might appear to meet the inclusion criteria, but which were excluded, and explain why they were excluded.	Not reported
Study characteristics	17	Cite each included study and present its characteristics.	Table S6
Risk of bias in studies	18	Present assessments of risk of bias for each included study.	Table S4
Results of individual studies	19	For all outcomes, present, for each study: (a) summary statistics for each group (where appropriate) and (b) an effect estimate and its precision (e.g. confidence/credible interval), ideally using structured tables or plots.	Table S6
Results of syntheses	20a	For each synthesis, briefly summarise the characteristics and risk of bias among contributing studies.	Results section under each surgical domain.
	20b	Present results of all statistical syntheses conducted. If meta-analysis was done, present for each the summary estimate and its precision (e.g. confidence/credible interval) and measures of statistical heterogeneity. If comparing groups, describe the direction of the effect.	Not applicable
	20c	Present results of all investigations of possible causes of heterogeneity among study results.	Not applicable
	20d	Present results of all sensitivity analyses conducted to assess the robustness of the synthesized results.	Not reported
Reporting	21	Present assessments of risk of bias due to missing results (arising from	Not reported

Section and Topic	Item #	Checklist item	Location where item is reported
biases		reporting biases) for each synthesis assessed.	
Certainty of evidence	22	Present assessments of certainty (or confidence) in the body of evidence for each outcome assessed.	Not reported
**DISCUSSION**			
Discussion	23a	Provide a general interpretation of the results in the context of other evidence.	Discussion
	23b	Discuss any limitations of the evidence included in the review.	Discussion
	23c	Discuss any limitations of the review processes used.	Discussion
	23d	Discuss implications of the results for practice, policy, and future research.	Discussion
**OTHER INFORMATION**			
Registration and protocol	24a	Provide registration information for the review, including register name and registration number, or state that the review was not registered.	Methods > Search strategy
	24b	Indicate where the review protocol can be accessed, or state that a protocol was not prepared.	Methods > Registered in PROSPERO (CRD420251042541) (https://www.crd.york.ac.uk/PROSPERO/view/CRD420251042541)
	24c	Describe and explain any amendments to information provided at registration or in the protocol.	Methods > Search strategy
Support	25	Describe sources of financial or non-financial support for the review, and the role of the funders or sponsors in the review.	Funding
Competing interests	26	Declare any competing interests of review authors.	Declaration of Interest
Availability of data, code and other materials	27	Report which of the following are publicly available and where they can be found: template data collection forms; data extracted from included studies; data used for all analyses; analytic code; any other materials used in the review.	Data availability statement

Table S3. PRISMA Abstract Checklist.

Section and Topic	Item #	Checklist item	Reported (Yes/No)
**TITLE**			
Title	1	Identify the report as a systematic review.	Yes
**BACKGROUND**			
Objectives	2	Provide an explicit statement of the main objective(s) or question(s) the review addresses.	Yes
**METHODS**			
Eligibility criteria	3	Specify the inclusion and exclusion criteria for the review.	Yes
Information sources	4	Specify the information sources (e.g. databases, registers) used to identify studies and the date when each was last searched.	Yes
Risk of bias	5	Specify the methods used to assess risk of bias in the included studies.	Yes
Synthesis of results	6	Specify the methods used to present and synthesise results.	Yes
**RESULTS**			
Included studies	7	Give the total number of included studies and participants and summarise relevant characteristics of studies.	Yes
Synthesis of results	8	Present results for main outcomes, preferably indicating the number of included studies and participants for each. If meta-analysis was done, report the summary estimate and confidence/credible interval. If comparing groups, indicate the direction of the effect (i.e. which group is favoured).	Yes
**DISCUSSION**			
Limitations of evidence	9	Provide a brief summary of the limitations of the evidence included in the review (e.g. study risk of bias, inconsistency and imprecision).	Yes
Interpretation	10	Provide a general interpretation of the results and important implications.	Yes
**OTHER**			

Section and Topic	Item #	Checklist item	Reported (Yes/No)
Funding	11	Specify the primary source of funding for the review.	Yes
Registration	12	Provide the register name and registration number.	Yes

Table S4. AMSTAR2 evaluation of the key studies in each surgical domain.

Domain	High	Moderate	Low	Critically Low	Number of Reviews
Esophageal	10	0	4	11	25
Gastric	0	30	10	4	44
Liver	16	11	1	5	33
Biliary	5	0	9	16	30
Pancreatic	15	2	14	9	40
Colorectal	27	22	19	10	78
Total	73 (29.2%)	65 (26.0%)	57 (22.8%)	55 (22.0%)	250

Table S5. CCA calculation using a citation matrix of eligible reviews in each surgical domain.

Domain	Number of Included Studies	Number of Unique Primary Studies	Number of Reviews	CCA Value	Overlap Level
Esophageal	164	52	25	8.97%	Moderate
Gastric	589	150	44	6.81%	Moderate
Liver	150	38	33	8.93%	Moderate
Biliary	119	101	30	0.61%	Slight
Pancreatic	449	231	40	0.02%	Slight
Colorectal	787	466	78	0.01%	Slight

Table S6. General characteristics of the 250 articles included in this umbrella review.

Author/Year	Study Design	Procedure Type	Surgical Approach	Key Findings	AMSTAR2 Rating
**ESOPHAGEAL SURGERY**					
McKinley et al.(2021)	Systematic review	Fundoplication	Robotic vs Laparoscopic	Similar outcomes between approaches; robotic longer operative time and higher cost.	Critically Low
Damani et al.(2020)	Review	Fundoplication, Heller myotomy	Robotic vs Laparoscopic	Robotic surgery showed fewer perforations and better performance in reoperations.	Critically Low
Fiume et al.(2022)	Review	Esophagomyotomy	Robotic vs Laparoscopic	Robotic myotomy had lower perforation risk and improved technical control.	Critically Low
Watanabe et al.(2023)	Review	Esophagectomy	Robotic vs Laparoscopic vs Open	RAMIE reduced pulmonary complications and improved lymph node dissection.	Critically Low
Rebecchi et al.(2023)	Review	Esophagectomy	Robotic vs Laparoscopic vs Open	Robotic surgery had better short-term outcomes but higher cost than conventional approaches.	Critically Low
Witek et al.(2020)	Review	Esophagectomy	Robotic vs Laparoscopic vs Open	Minimally invasive techniques reduce morbidity; approach choice depends on surgeon expertise.	Critically Low
Zhou et al.(2022)	Systematic review	Esophagectomy	Robotic vs Laparoscopic	Robotic surgery reduced pulmonary complications, increased lymph node harvest.	Low
Ramjit et al.(2022)	Systematic review	Esophagectomy	Robotic vs Open	MIE improved safety and similar efficacy; limited data on cost-effectiveness.	High
Kanamori et al.(2022)	Review	Esophagectomy	Robotic vs Laparoscopic vs	Robotic surgery enhances left upper mediastinal lymph node dissection.	Critically Low

			Open		
Angeramo et al.(2021)	Systematic review	Esophagectomy	Robotic vs Laparoscopic	Robotic surgery reduced blood loss, pulmonary complications, improved R0 resection rate.	Low
Aiolfi et al.(2025)	Systematic review	Esophagomyotomy	Robotic vs Laparoscopic	Robotic surgery reduced perforation risk with comparable functional outcomes.	High
Jin et al.(2019)	Systematic review	Esophagectomy	Robotic vs Laparoscopic	Robotic surgery reduced blood loss and vocal cord palsy rates.	Low
Li et al.(2021)	Systematic review	Esophagectomy	Robotic vs Laparoscopic	Robotic approach yielded higher lymph node harvest and reduced vocal cord palsy.	Low
Weindelmayer et al.(2023)	Review	Esophagectomy	Robotic vs Open	Robotic approach retrieved more lymph nodes with similar complication rates.	Critically Low
Siaw-Acheampong et al.(2020)	Systematic review	Esophagectomy	Robotic vs Laparoscopic vs Open	Minimally invasive approaches reduced morbidity and hospital stay without compromising oncologic outcomes.	High
Milone et al.(2019)	Systematic review	Heller myotomy	Robotic vs Laparoscopic	Robotic surgery lowered perforation risk with similar outcomes.	High
Xie et al.(2021)	Systematic review	Heller myotomy	Robotic vs Laparoscopic	Robotic approach had fewer esophageal perforations than laparoscopic.	High
Patel et al.(2024)	Systematic review	Esophagectomy	Robotic vs Open	Robotic esophagectomy improved pneumonia and leak rates, with high R0 resection.	High
Mederos et al.(2021)	Systematic review	Esophagectomy	Robotic vs Laparoscopic vs Open	Robotic approach reduced pulmonary complications; similar outcomes otherwise.	High
Gonçalves-Costa et al.(2024)	Systematic review	Fundoplication, Hiatal hernia repair	Robotic vs Laparoscopic	Outcomes were comparable; laparoscopic was more cost-effective.	High
Huang et	Systematic	Esophagectomy	Robotic vs	Robotic surgery had less blood loss and	High

al.(2021)	review		Laparoscopic	fewer pulmonary complications.	
Perry et al.(2024)	Systematic review	Esophagectomy	Robotic vs Laparoscopic	Robotic surgery showed better blood loss, lymph node yield, and shorter stays.	High
Vashist et al.(2025)	Review	Esophagectomy	Robotic vs Open	Robotic surgery reduced complications; multicenter validation required.	Critically Low
Tartaglia et al.(2020)	Review	Hiatal hernia	Robotic vs Laparoscopic	Robotic approach effective with low morbidity in complex hernia cases.	Critically Low
Karikis et al.(2024)	Review	Hiatal hernia	Robotic vs Laparoscopic	Robotic surgery reduced complications and improved visualization.	Critically Low
**GASTRIC SURGERY**					
Guerrini et al.(2020)	Meta-analysis	Gastrectomy	Robotic vs Laparoscopic	Robotic reduced blood loss, complication rates, and improved lymph node harvest.	Low
Shibasaki et al.(2023)	Systematic review	Gastrectomy	Robotic vs Laparoscopic	Robotic had lower blood loss and shorter hospital stay but higher cost.	Low
Zizzo et al.(2022)	Systematic review	Gastrectomy	Robotic vs Laparoscopic	Robotic improved lymph node harvest and reduced blood loss.	Moderate
van Boxel et al.(2019)	Review	Gastrectomy	Robotic vs Laparoscopic	Robotic reduced blood loss with similar survival outcomes.	Low
Shibasaki et al.(2020)	Review	Gastrectomy	Robotic vs Laparoscopic vs Open	Robotic showed less blood loss, faster recovery, but higher cost.	Low
Zhang et al.(2021)	Systematic review	Bariatric surgery	Robotic vs Laparoscopic	Robotic prolonged operative time but had lower mortality.	Low
Roriz-Silva et al.(2022)	Review	Gastric bypass	Robotic vs Laparoscopic	Robotic increased operative time; similar outcomes at 12 months.	Low
Ong et al.(2022)	Review	Gastrectomy	Robotic vs Laparoscopic vs	Robotic reduced blood loss and improved lymph node harvest.	Low

			Open		
Chen et al.(2022)	Meta-analysis	Gastrectomy	Robotic vs Open	Robotic reduced complications, blood loss, and hospital stay.	Moderate
Kim et al.(2021)	Review	Gastrectomy	Robotic vs Laparoscopic	Robotic reduced blood loss and had comparable oncologic outcomes.	Low
Bertoni et al.(2021)	Systematic review	Revisional bariatric surgery	Robotic vs Laparoscopic	Robotic had no significant advantage over laparoscopic in perioperative outcomes.	Moderate
Ma et al.(2020)	Systematic review	Gastrectomy	Robotic vs Laparoscopic	Robotic showed less blood loss, similar survival, and longer operative time.	Moderate
Wang et al.(2018)	Systematic review	Gastric bypass	Robotic vs Laparoscopic	Robotic and laparoscopic had similar outcomes; robotic had longer operative time.	Moderate
Marano et al.(2021)	Review	Gastrectomy	Robotic vs Laparoscopic	Robotic improved lymph node retrieval; operative time longer than laparoscopic.	Moderate
Davey et al.(2023)	Systematic review	Gastrectomy	Robotic vs Laparoscopic vs Open	Robotic and laparoscopic superior to open in recovery; outcomes were similar.	Moderate
Zhao et al.(2024)	Systematic review	Sleeve gastrectomy	Robotic vs Laparoscopic	Robotic had similar efficacy but longer hospital stay and higher cost.	Moderate
Morrell et al.(2021)	Review	Gastric bypass	Robotic	Robotic was feasible and safe with low complication rates in short term.	Critically Low
Kotidis et al.(2019)	Review	Gastrectomy with D2 lymphadenectomy	Robotic vs Laparoscopic	Robotic improved lymph node retrieval; long-term outcomes still debated.	Critically Low
Gong et al.(2022)	Systematic review	Distal gastrectomy	Robotic vs Laparoscopic	Robotic reduced blood loss and complications; longer operative time.	Moderate
Guerra et al.(2018)	Systematic review	Radical gastrectomy	Robotic vs Laparoscopic	Robotic showed lower pancreatic complication rate, though not statistically significant.	Moderate

Li et al.(2024)	Systematic review	Gastrectomy	Robotic vs Laparoscopic	Robotic improved OS, reduced complications and blood loss, but had longer operative time.	Moderate
Leang et al.(2024)	Systematic review	Gastric bypass	Robotic vs Laparoscopic	No significant difference in outcomes; robotic had higher reoperation rate.	Moderate
Aoyama et al.(2024)	Review	Distal gastrectomy	Robotic vs Laparoscopic vs Open	Robotic showed better QOL and equivalent oncologic outcomes compared to laparoscopic.	Moderate
Du et al.(2025)	Systematic review	Gastrectomy	Robotic vs Laparoscopic	Robotic reduced complications, blood loss, and hospital stay but increased cost and time.	Moderate
Triemstra et al.(2024)	Systematic review	Minimally invasive gastrectomy	Robotic vs Laparoscopic	Robotic showed fewer complications and similar survival, but higher cost.	Low
Kossenas et al.(2024)	Systematic review	Distal gastrectomy	Robotic vs Laparoscopic	Robotic had faster recovery, similar safety, and longer operative time.	Moderate
Aiolfi et al.(2019)	Systematic review	Gastric bypass	Robotic vs Laparoscopic vs Open	Robotic and laparoscopic had fewer complications and lower mortality than open.	Moderate
Solaini et al.(2020)	Systematic review	Gastrectomy	Robotic vs Laparoscopic vs Open	Robotic had less blood loss, similar survival, longer operative time, and higher cost.	Moderate
Qiu et al.(2019)	Systematic review	Gastrectomy	Robotic vs Laparoscopic	Robotic reduced blood loss and increased cost and time, with similar survival.	Moderate
Hoshino et al.(2020)	Review	Gastrectomy	Robotic vs Laparoscopic	Robotic showed less blood loss and faster oral intake; overall evidence quality was low.	Moderate
Wang et al.(2025)	Meta-analysis	Gastrectomy	Robotic vs Laparoscopic	Robotic had less blood loss and quicker recovery; fewer lymph nodes retrieved.	Moderate
Sun et al.(2022)	Meta-analysis	Distal gastrectomy	Robotic vs	Robotic surgery had less blood loss,	Moderate

			Laparoscopic	more lymph nodes, but longer operative time.	
Du et al.(2024)	Systematic review	Gastric bypass	Robotic vs Laparoscopic	Robotic reduced long-term complications; no difference in major outcomes; higher cost.	Moderate
Aiolfi et al.(2021)	Systematic review	Distal gastrectomy	Robotic vs Laparoscopic vs Open	Robotic showed less blood loss and faster recovery; similar survival outcomes.	Moderate
Li et al.(2024)	Meta-analysis	Distal gastrectomy	Robotic vs Laparoscopic	Robotic improved lymph node yield and hospital stay, but prolonged operative time.	Moderate
Jin et al.(2022)	Meta-analysis	Gastrectomy	Robotic vs Laparoscopic	Robotic showed fewer complications and more lymph nodes; long-term outcomes similar.	Moderate
Zheng et al(2019)	Meta-analysis	Gastrectomy	Robotic vs Laparoscopic	Robotic had lower blood loss and complications; similar survival outcomes.	Moderate
Zhang et al.(2021)	Meta-analysis	Distal subtotal gastrectomy	Robotic vs Laparoscopic	Robotic had less blood loss, more lymph nodes; similar complication rates.	Low
Komatsu et al.(2023)	Review	Gastrectomy	Robotic vs Laparoscopic	Robotic reduced complications in overweight patients; particularly grade ≥III complications.	Critically Low
Yang et al.(2024)	Meta-analysis	Gastrectomy	Robotic vs Laparoscopic	Robotic reduced major complications; operative time was longer.	Moderate
Marano et al.(2025)	Review	gastrectomy	Robotic vs Laparoscopic	Robotic gastrectomy shows less blood loss and morbidity, but longer operation time and higher cost.	Critically Low
Kossenas et al.(2025)	Systematic review and meta-analysis	total gastrectomy	Robotic vs Laparoscopic	Comparable safety outcomes; robotic approach viable where available.	Moderate

Liao et al.(2019)	Meta-analysis	gastrectomy	Robotic vs Laparoscopic	No significant difference in survival or recurrence; long-term outcomes comparable.	Moderate
Huang et al.(2025)	Meta-analysis	gastrectomy	Robotic vs Laparoscopic	Robotic approach associated with reduced complications, blood loss, and improved recovery metrics.	Moderate
**LIVER SURGERY**					
Benedetto et al.(2020)	Review	Liver resection	Robotic vs Laparoscopic vs Open	Robotic liver surgery offers safety and feasibility with improved short-term outcomes and comparable oncologic results.	Critically Low
Hu et al.(2021)	Systematic review and meta-analysis	Hepatectomy for malignancy	Robotic vs Laparoscopic	Robotic surgery had lower conversion and higher right hepatectomy rate; outcomes otherwise similar.	Moderate
Ayabe et al.(2022)	Review	Liver resection	Robotic vs Laparoscopic	Shorter learning curve, better for complex resections; limitations include higher cost and limited instruments.	Critically Low
Xuea et al.(2023)	Systematic review and meta-analysis	Hepatectomy	Robotic vs Open	Robotic surgery associated with less blood loss, fewer complications, and shorter hospital stay than open surgery.	High
Ziogas et al.(2021)	Systematic review and meta-analysis	Major hepatectomy	Robotic vs Laparoscopic	Similar outcomes between robotic and laparoscopic approaches for major hepatectomy.	High
Kamarajah et al.(2021)	Systematic review and meta-analysis	Liver resection	Robotic vs Laparoscopic	Robotic surgery showed lower readmission rates but longer operating time than laparoscopic surgery.	Low
Guan et al.(2019)	Meta-analysis	Liver resection	Robotic vs Laparoscopic	Robotic resection had longer operative time and higher cost, with similar safety and effectiveness.	Critically Low
Becker et al.(2021)	Review	Liver resection	Robotic vs	Robotic surgery advantageous in	Critically Low

			Laparoscopic vs Open	complex cases and a viable alternative to open surgery.	
Wang et al.(2021)	Systematic review and meta-analysis	Minor hepatectomy	Robotic vs Laparoscopic	Similar safety and effectiveness; robotic approach had longer operative time and higher cost.	High
Koh et al.(2024)	Systematic review and network meta-analysis	Liver resection	Robotic vs Laparoscopic vs Open	Laparoscopic most cost-effective; robotic offered reduced morbidity and shorter hospital stay but highest cost.	High
Wong et al.(2019)	Systematic review	Hepatectomy	Robotic vs Open	Robotic had lower complications and shorter stay, but longer operative time.	Moderate
Magistri et al.(2019)	Systematic review	Hepatectomy for HCC	Robotic vs Laparoscopic vs Open	Robotic showed effective oncologic outcomes in experienced centers.	Moderate
Coletta et al.(2021)	Systematic review	Major hepatectomy	Robotic vs Laparoscopic	Robotic had less blood loss and conversion; laparoscopy had shorter hospital stay.	Moderate
Gavriilidis et al.(2020)	Systematic review	Hepatectomy	Robotic vs Laparoscopic vs Open	Robotic and laparoscopic reduced morbidity and hospital stay compared to open.	High
Machairas et al.(2019)	Systematic review	Hepatectomy	Robotic vs Open	Robotic had lower morbidity and shorter stay, but longer operative time.	High
Ziogas et al.(2021)	Systematic review	Hepatectomy	Robotic vs Laparoscopic vs Open	Robotic had highest cost; laparoscopic had lowest hospitalization cost.	Moderate
He et al.(2024)	Meta-analysis	Hepatectomy	Robotic vs Open	Robotic reduced complications and blood loss but fewer major resections performed.	High
Mao et al.(2023)	Systematic review	Major hepatectomy	Robotic vs Laparoscopic	Robotic showed lower complications, conversion, and blood loss.	Critically Low

Gao et al.(2023)	Meta-analysis	Liver resection	Robotic vs Laparoscopic	Robotic had less blood loss, conversion, and severe complications.	High
Hu et al.(2018)	Meta-analysis	Hepatectomy	Robotic vs Laparoscopic	Robotic had more blood loss and longer time; similar safety.	Moderate
Long et al.(2024)	Systematic review	Liver resection for malignancy	Robotic vs Laparoscopic	Comparable surgical and oncologic outcomes; no clear superiority of robotic.	High
Wang et al.(2025)	Meta-analysis	Hepatectomy	Robotic vs Laparoscopic	Robotic showed lower blood loss, fewer conversions, and higher R0 resection.	Moderate
Del Angel Millan et al.(2024)	Systematic review	Caudate lobectomy	Robotic	Robotic caudatectomy is safe and feasible with low complication and conversion rates.	High
Troisi et al.(2020)	Review	Liver resection	Robotic vs Laparoscopic	Robotic offers ergonomic and precision advantages; long-term outcomes remain unclear.	High
Hajibandeh et al.(2022)	Meta-analysis	Left lateral sectionectomy	Robotic vs Laparoscopic	Robotic had longer procedure time and higher cost; outcomes otherwise similar.	Moderate
Yeow et al.(2022)	Systematic review	Living donor right hepatectomy	Robotic vs Laparoscopic vs Open	Robotic had longer time, less blood loss; overall complications comparable.	High
Zhang et al.(2020)	Meta-analysis	Liver resection	Robotic vs Laparoscopic	Robotic had longer time, lower conversion rate, higher cost.	High
Giannone et al.(2025)	Systematic review	Posterosuperior segmentectomy	Robotic vs Laparoscopic	Robotic had less blood loss, fewer transfusions, and shorter operative time.	Moderate
Ciria et al.(2022)	Systematic review	Liver resection	Robotic vs Laparoscopic vs Open	Robotic had some short-term advantages; cost-effectiveness remains controversial.	Moderate
Niu et al.(2025)	Meta-analysis	Hepatectomy for liver tumors	Robotic vs Laparoscopic	Robotic had longer time and cost; overall outcomes similar.	High

Gheza et al.(2019)	Systematic review	Robotic hepatectomy	Robotic	Conversion mainly due to bleeding and margin assessment; robotic reduces adhesion-related conversions.	High
Zhang et al.(2022)	Meta-analysis	Hepaticojejunostomy in pediatric CDD	Robotic vs Laparoscopic	Robotic had fewer complications, shorter stay, and less bleeding, but higher cost.	Moderate
Linn et al.(2023)	Systematic review	Liver resection (all types)	Robotic vs Laparoscopic	Validated difficulty scores predict complexity; robotic-specific scoring needs further research.	High
**BILIARY SURGERY**					
Chang et al.(2020)	Review	Cholecystectomy, CBD exploration	Robotic vs Laparoscopic	Robotic aids complex dissection, improves visualization, but increases cost.	Critically low
Straatman et al.(2023)	Systematic review	Multiport cholecystectomy	Robotic vs Laparoscopic	Robotic increases operative time; conversion rate lower; other outcomes similar.	Low
Wang et al.(2021)	Systematic review	Single-incision cholecystectomy	Robotic vs Laparoscopic	Robotic had lower complication and hernia rates but higher cost.	Critically low
Delgado et al.(2024)	Systematic review	Cholecystectomy for benign disease	Robotic vs Laparoscopic	Robotic increased operative time; other perioperative outcomes similar.	High
Cubisino et al.(2023)	Systematic review	Biliary anastomosis for BDI	Robotic vs Laparoscopic	Robotic had no conversions and similar morbidity; feasibility demonstrated.	Critically low
Sanford(2019)	Review	Cholecystectomy	Robotic vs Laparoscopic	Robotic safe but more expensive and time-consuming; good for surgeon training.	Critically low
Wang et al.(2021)	Systematic review	Hilar cholangiocarcinoma resection	Robotic vs Laparoscopic	Robotic had less conversion; higher complication rates; promising but early.	Critically low
Chee et al.(2024)	Systematic review	Gallbladder cancer resection	Robotic vs Laparoscopic vs Open	No major survival differences; robotic offered shorter stays and less blood loss.	Critically low

Sun et al.(2018)	Meta-analysis	Single-incision cholecystectomy	Robotic vs Laparoscopic	Outcomes similar; robotic had higher cost but same safety and feasibility.	Low
Han et al.(2018)	Meta-analysis	Cholecystectomy for benign disease	Robotic vs Laparoscopic	Robotic had longer time and higher hernia risk; overall outcomes comparable.	Low
Sun et al.(2018)	Systematic review	Cholecystectomy	Robotic vs Laparoscopic	Robotic had higher incisional hernia and cost; other outcomes equivalent.	Critically low
Migliore et al.(2018)	Systematic review	Single-incision cholecystectomy	Robotic	Robotic showed low complication and conversion rates; port site hernia noted.	Critically low
Shenoy et al.(2021)	Systematic review	Cholecystectomy	Robotic vs Laparoscopic	Outcomes similar; robotic had longer OR time and higher hernia in some cases.	Low
Dong et al.(2024)	Systematic review	Cholangiocarcinoma	Robotic vs Laparoscopic vs Open	Robotic had better R0, complication, and transfusion rates than others.	Low
Wang et al.(2024)	Systematic review	Gallbladder cancer	Robotic vs Open	Robotic had less blood loss, shorter stay, fewer complications.	Low
Hu et al.(2025)	Systematic review	Hilar cholangiocarcinoma	Robotic vs Open	Outcomes similar; robotic safe and feasible with no statistical superiority.	High
Liu et al.(2021)	Review	Gallbladder carcinoma	Robotic vs Laparoscopic	MIS safe in selected cases; robotic has good R0 rate and low complications.	Critically low
Lin et al.(2023)	Systematic review	Cholecystectomy	Robotic vs Laparoscopic	Robotic best for pain relief and hospital stay; laparoscopic better for complications.	Low
Wu et al.(2024)	Systematic review	Cholangiocarcinoma	Robotic vs Open	Robotic had less blood loss, shorter stay; other outcomes similar.	High
Cipriani et al.(2021)	Systematic review	Hilar cholangiocarcinoma	Robotic vs Laparoscopic	MIS safe and effective in selected cases; robotic outcomes promising.	Critically low
Nam et al.(2024)	Review	Cholecystectomy	Robotic vs	Robotic offers improved dexterity;	Critically low

			Laparoscopic	further studies needed on safety and efficacy.	
Hu et al.(2019)	Review	Hilar cholangiocarcinoma	Robotic vs Laparoscopic	Robotic feasible in selected patients; limited evidence and high morbidity.	Critically low
Kirkham et al.(2022)	Systematic review	Cholecystectomy	Robotic	IDEAL guidelines poorly followed; many studies lacked rigorous evaluation.	Critically low
Mellado et al.(2024)	Review	Gallbladder cancer	Robotic vs Laparoscopic vs Open	Robotic safe and effective; requires more data to confirm oncologic safety.	Critically low
Franken et al.(2019)	Systematic review	Perihilar cholangiocarcinoma	Robotic vs Laparoscopic vs Open	Robotic feasible in experienced centers; limited comparative data available.	Critically low
Jensen et al.(2021)	Systematic review	Cholecystectomy	Robotic vs Laparoscopic vs Open	Robotic and SILC showed higher hernia rates than multiport laparoscopy.	Low
Pacilli et al.(2024)	Review	Subtotal cholecystectomy	Robotic vs Laparoscopic	Robotic useful in complex cases; enhances safety in severe cholecystitis.	Critically low
Kossenas et al.(2024)	Systematic review	Cholecystectomy	Robotic vs Laparoscopic	Robotic had longer duration; shorter hospital stay; similar complications.	Low
Tang et al.(2021)	Systematic review	Hilar cholangiocarcinoma	Robotic vs Open	Comparable outcomes; robotic had less blood loss and shorter stay.	High
Zhang et al.(2024)	Systematic review	Kasai portoenterostomy	Robotic vs Laparoscopic vs Open	Robotic had higher COJ rates; no statistical difference between techniques.	High
**PANCREATIC SURGERY**					
Chaouch et al.(2023)	Systematic review	Total pancreatectomy	Robotic vs Open	Robotic had similar outcomes but enabled quicker recovery and oral intake.	High
van Ramshorst et	Systematic	Distal pancreatectomy	Robotic vs	Robotic had lower conversion rate,	High

al.(2023)	review		Laparoscopic	higher cost, and higher lymph node yield in PDAC.	
Mavrovounis et al.(2020)	Systematic review	Peripheral pancreatectomy	Robotic vs Laparoscopic	Robotic had longer time, better spleen preservation, and higher cost.	Low
Rompianesi et al.(2022)	Systematic review	Central pancreatectomy	Robotic	Robotic safe with low mortality, but high pancreatic fistula and complication rates.	High
Kamarajah et al.(2020)	Systematic review	Pancreaticoduodenectomy	Robotic vs Laparoscopic	Robotic had lower conversion and transfusion rates; other outcomes were similar.	High
Mantzavinou et al.(2022)	Systematic review	Pancreaticoduodenectomy	Robotic vs Open	Robotic had higher therapeutic index and lower mortality; oncologic outcomes similar.	Low
Kamarajah et al.(2019)	Systematic review	Distal pancreatectomy	Robotic vs Laparoscopic	Robotic had longer time but lower conversion rates and shorter hospital stay.	Critically Low
Farrarons et al.(2022)	Systematic review	Central pancreatectomy	Robotic vs Open	Robotic had fewer transfusions and better function; fistula rates were similar.	Low
Khachfe et al.(2022)	Review	Pancreatic resections (various)	Robotic vs Laparoscopic vs Open	Robotic is promising but high cost and learning curve remain concerns.	Critically Low
Roesel et al.(2023)	Systematic review	Pancreatic enucleation	Robotic vs Laparoscopic vs Open	Robotic safe and effective; reduced hospital stay with similar complications.	Low
Dalla Valle et al.(2019)	Systematic review	Pancreatic enucleation	Robotic vs Open	Minimally invasive approach showed significantly lower morbidity and POPF rates.	Critically Low
Li et al.(2023)	Systematic review	Distal pancreatectomy	Robotic vs Laparoscopic	Robotic reduced conversion, improved spleen preservation, but increased cost.	Low

Lee et al.(2023)	Systematic review	Distal pancreatectomy, pancreaticoduodenectomy	Robotic vs Open	MIS increased OR cost but had similar overall index hospitalization cost.	Low
Neshan et al.(2024)	Systematic review	Pancreaticoduodenectomy	Robotic vs Open	Robotic had better survival and shorter stay; costs were higher.	High
Zhang et al.(2021)	Systematic review	Pancreaticoduodenectomy	Robotic vs Open	Robotic reduced EBL, complications, and transfusions; longer operative time.	Low
Da Dong et al.(2021)	Systematic review	Pancreaticoduodenectomy	Robotic vs Open	Robotic improved R0 rate and lymph node yield; longer operative time.	High
Aiolfi et al.(2021)	Systematic review	Pancreaticoduodenectomy	Robotic vs Laparoscopic vs Open	Robotic and laparoscopic had fewer infections and shorter stay than open.	High
Kamarajah et al.(2020)	Systematic review	Pancreaticoduodenectomy	Robotic vs Laparoscopic vs Open	Robotic had lower transfusion and infection rates; longer operative time.	High
Zhou et al.(2020)	Systematic review	Distal pancreatectomy	Robotic vs Open	Robotic had less blood loss, mortality, and shorter stay; other outcomes similar.	Critically Low
Uijterwijk et al.(2023)	Systematic review	Pancreaticoduodenectomy	Robotic vs Open	Minimally invasive had less blood loss and shorter stay; oncologic outcomes similar.	High
Shyr et al.(2020)	Review	Pancreaticoduodenectomy	Robotic vs Open	Robotic had less blood loss, shorter stay, and high patient satisfaction in experienced hands.	Critically Low
Gavriilidis et al.(2019)	Systematic review	Distal pancreatectomy	Robotic vs Laparoscopic vs Open	Robotic and laparoscopic showed less blood loss and shorter stay than open.	Critically Low
Kabir et al.(2022)	Systematic review	Pancreaticoduodenectomy	Robotic vs Laparoscopic vs Open	Robotic had least blood loss and fewer infections; laparoscopic had shortest stay.	Low
Partelli et al.(2021)	Systematic review	Distal pancreatectomy	Robotic vs Laparoscopic vs	Robotic safest; laparoscopic most cost-efficient; open had lowest procedure	Low

			Open	cost.	
Di Martino et al.(2021)	Systematic review	Distal pancreatectomy	Robotic vs Laparoscopic	Robotic had lower conversion rate and higher cost; similar outcomes otherwise.	Critically Low
Wang et al.(2023)	Systematic review	Pancreaticoduodenectomy	Robotic vs Laparoscopic vs Open	Robotic had lowest risk of pancreatic fistula; both MIS better than open.	Low
Yan et al.(2020)	Systematic review	Pancreaticoduodenectomy	Robotic vs Open	Robotic had less blood loss, lower wound infection, shorter stay, but longer time.	Low
Podda et al.(2020)	Systematic review	Pancreaticoduodenectomy	Robotic vs Open	Robotic and open had similar outcomes; robotic had less blood loss.	High
Armengol-García et al.(2025)	Systematic review	Pancreaticoduodenectomy	Robotic vs Laparoscopic	Robotic had fewer conversions and higher lymph node yield.	High
Zhao et al.(2018)	Systematic review	Pancreaticoduodenectomy, Distal pancreatectomy	Robotic vs Open	Robotic had less blood loss, fewer infections, similar mortality and lymph node yield.	Critically Low
Tang et al.(2025)	Systematic review	Pancreaticoduodenectomy	Robotic vs Laparoscopic	Robotic had lower conversion, transfusion rates, and more lymph nodes retrieved.	High
Xu et al.(2025)	Systematic review	Distal pancreatectomy	Robotic vs Laparoscopic	Robotic had better conversion and blood loss, but longer operative time.	Low
Hu et al.(2020)	Systematic review	Distal pancreatectomy	Robotic vs Laparoscopic	Robotic had higher spleen preservation but higher cost; other outcomes similar.	Critically Low
Tang et al.(2025)	Systematic review	Pancreaticoduodenectomy	Robotic vs Open	Robotic had less blood loss, lower transfusion and infection rates, shorter stay.	High
Lyu et al.(2021)	Systematic review	Distal pancreatectomy	Robotic vs Laparoscopic vs Open	Robotic had least complications, shortest stay, and best lymph node yield.	Low

Niu et al.(2019)	Systematic review	Distal pancreatectomy	Robotic vs Laparoscopic vs Open	Robotic had shorter stay and lower complications; longer operative time.	High
Luo et al.(2024)	Systematic review	Pancreaticoduodenectomy	Robotic vs Open	Robotic had shorter stay, less blood loss, but higher reoperation rate.	Low
Xu et al.(2019)	Systematic review	Distal pancreatectomy	Robotic vs Laparoscopic	Robotic had better vessel preservation; operative cost significantly higher.	Moderate
Koh et al.(2024)	Systematic review	Distal pancreatectomy	Robotic vs Laparoscopic vs Open	Robotic had highest cost but best surgical outcomes and QALYs.	High
Kossenas et al.(2025)	Systematic review	Pancreaticoduodenectomy	Robotic vs Laparoscopic	Robotic had fewer complications, shorter stay, and better lymph node harvest.	Moderate
**COLORECTAL SURGERY**					
Tang et al.(2021)	Meta-analysis	Rectal cancer surgery	Robotic vs Laparoscopic	Similar complication rates; robotic had slightly lower conversion rate, longer operative time.	Moderate
Cuk et al.(2022)	Systematic review	Colon cancer resection	Robotic vs Laparoscopic	Robotic had lower conversion and complication rates; longer operative time.	High
Tong et al.(2021)	Meta-analysis	Rectal cancer surgery	Robotic vs Laparoscopic	Robotic had better pelvic visibility; comparable short- and long-term outcomes.	Critically Low
Solaini et al.(2022)	Systematic review	Left colectomy	Robotic vs Laparoscopic	Robotic had lower conversion and complication rates; longer operative time.	High
McKechnie et al.(2025)	Systematic review	Colorectal surgery (obesity)	Robotic vs Laparoscopic	Robotic reduced conversion to laparotomy; no difference in morbidity; longer operative time.	High
Fleming et	Meta-analysis	Rectal cancer surgery	Robotic vs	Robotic preserved urinary and erectile	High

al.(2021)			Laparoscopic	function better in male patients.	
Grass et al.(2021)	Systematic review	Rectal cancer surgery	Robotic vs Laparoscopic vs Open	Robotic had significantly better anorectal function post-op than other approaches.	Low
Sun et al.(2019)	Systematic review	Rectal cancer surgery	Robotic vs Laparoscopic	Robotic had lower morbidity and conversion rate; similar oncologic outcomes.	Low
Wang et al.(2020)	Systematic review	Rectal cancer surgery	Robotic vs Laparoscopic	Robotic had lower complications, conversion rate, and faster bowel recovery.	Critically Low
Solaini et al.(2018)	Systematic review	Right colectomy	Robotic vs Laparoscopic	Robotic had fewer conversions and shorter recovery; higher cost.	Low
Flynn et al.(2021)	Systematic review	Ventral mesh rectopexy	Robotic vs Laparoscopic	Robotic reduced length of stay; similar complications and conversion rates; longer operating time.	Low
Holmer et al.(2018)	Systematic review	Low anterior resection	Robotic vs Laparoscopic vs Open	Robotic had fewer conversions and transfusions; similar oncologic outcomes.	Critically Low
Flynn et al.(2021)	Systematic review	IPAA	Robotic vs Laparoscopic	Robotic had lower blood loss and shorter stay; similar complications and functional outcomes.	Low
Zelhart et al.(2018)	Review	Colorectal surgery	Robotic vs Laparoscopic vs Open	Robotic suitable for deep pelvis and complex tasks; limited data on cost-effectiveness.	Critically Low
Lee et al.(2018)	Systematic review	Intersphincteric resection	Robotic vs Laparoscopic	Robotic had lower conversion and blood loss; similar oncologic outcomes.	Low
Meyer et al.(2024)	Systematic review	Right hemicolectomy	Robotic vs Laparoscopic	Robotic had shorter stay, fewer conversions; higher lymph node yield.	Critically Low
Lam et al.(2021)	Review	Rectal cancer surgery	Robotic vs Laparoscopic	Similar margins, TME grade, lymph node yield, and survival; surgeon	Critically Low

				preference key.	
Kowalewski et al.(2021)	Meta-analysis	Rectal cancer resection	Robotic vs Laparoscopic	Robotic had better urinary outcomes and quality of life; sexual function similar.	High
Genova et al.(2021)	Systematic review	Right colectomy	Robotic vs Laparoscopic	Robotic had shorter stay, lower complications with IA; higher cost and operative time.	Moderate
Rubinkiewicz et al.(2019)	Systematic review	Rectal surgery	Robotic vs Laparoscopic	Robotic had better mesorectal excision and lower leakage; overall outcomes similar.	Moderate
Arang et al.(2023)	Systematic review	Appendicectomy	Robotic	Robotic appendicectomy is feasible and safe but limited by high cost and access.	Moderate
Yao et al.(2023)	Meta-analysis	Mid–low rectal cancer	Robotic vs Laparoscopic	Robotic had less blood loss, shorter stay, lower conversion; longer operative time.	Moderate
Gahunia et al.(2025)	Meta-analysis	Colorectal cancer (high-risk patients)	Robotic vs Laparoscopic	Robotic showed lower conversion rates and shorter stay in obese and male patients.	High
Li et al.(2025)	Meta-analysis	Rectal cancer (obese patients)	Robotic vs Laparoscopic	Robotic had shorter stay, fewer complications, lower readmission; longer OR time.	High
Morini et al.(2025)	Meta-analysis	Transverse colon cancer	Robotic vs Laparoscopic	Robotic had longer OR time but shorter stay; similar overall outcomes.	Moderate
Ng et al.(2019)	Meta-analysis	Colorectal cancer	Robotic vs Laparoscopic	Robotic had less conversion, less blood loss, shorter stay; longer OR time.	High
Albayati et al.(2019)	Systematic review	Ventral mesh rectopexy	Robotic vs Laparoscopic	Robotic showed longer OR time; similar morbidity, conversion, recurrence.	Moderate
Rausa et al.(2019)	Meta-analysis	Right hemicolectomy	Robotic vs Laparoscopic vs Open	RRH and TLRH had better short-term outcomes than LRH and ORH.	High
Giuliani et	Meta-analysis	Left-sided diverticular	Robotic vs	Robotic had lower conversion and	Moderate

al.(2022)		disease	Laparoscopic	shorter stay; longer operative time.	
Prete et al.(2018)	Meta-analysis	Rectal cancer	Robotic vs Laparoscopic	Robotic had lower conversion rates; similar margins, longer OR time.	High
Khan et al.(2024)	Meta-analysis	Rectal cancer	Robotic vs Laparoscopic	Robotic had lower conversion, lower CRM positivity, and lower reoperation rates.	High
Waters et al.(2020)	Systematic review	Right hemicolectomy	Robotic vs Laparoscopic	Robotic had shorter LOS, lower conversion, and higher lymph node yield.	Low
Wang et al.(2025)	Meta-analysis	Colorectal cancer (older patients)	Robotic vs Laparoscopic	Similar outcomes, but robotic had lower readmission and mortality rates.	High
Hoshino et al.(2019)	Systematic review	Rectal cancer	Robotic vs Laparoscopic	Robotic showed lower conversion rates; other benefits unclear due to review quality.	Low
Abdelsamad et al.(2024)	Meta-analysis	Extended mesorectal excision	Robotic vs Laparoscopic	Robotic had lower recurrence, fewer urinary complications, longer operative time.	Moderate
Simillis et al.(2019)	Network meta-analysis	Rectal cancer	Robotic vs Laparoscopic vs Open vs Transanal	Robotic had less blood loss, shorter stay, but longer OR time.	Moderate
Zaman et al.(2024)	Meta-analysis	IBD surgery	Robotic vs Laparoscopic	Robotic had fewer complications and shorter LOS; similar conversion and mortality.	High
Qiu et al.(2020)	Meta-analysis	Rectal cancer	Robotic vs Laparoscopic	Robotic and laparoscopic had comparable OS and DFS outcomes.	Moderate
Sheng et al.(2018)	Network meta-analysis	Colorectal cancer	Robotic vs Laparoscopic vs Open	Robotic had least bleeding, shortest stay, lowest complications among approaches.	Moderate
Oweira et al.(2023)	Meta-analysis	Colon cancer (CME)	Robotic vs Laparoscopic	Robotic had lower conversion, more lymph node harvest than laparoscopic	High

				non-CME.	
Geitenbeek et al.(2023)	Systematic review	Total mesorectal excision	Robotic vs Laparoscopic vs Open vs Transanal	Robotic had higher operative and lower hospital costs than others; overall cost-effectiveness remains inconclusive.	Moderate
Gonçalves et al.(2024)	Meta-analysis	Colon cancer surgery	Robotic vs Laparoscopic	Both approaches were similar in complications and hospital stay; robotic had longer time but faster recovery in some outcomes.	High
Chok et al.(2023)	Network meta-analysis	Colorectal surgery	Robotic vs Laparoscopic vs Open	Robotic had highest cost and best mortality outcome; laparoscopic was most cost-effective overall.	High
Ma et al.(2019)	Meta-analysis	Right colectomy	Robotic vs Laparoscopic	Robotic reduced complications and conversion but increased operative time.	Critically Low
Zou et al.(2025)	Meta-analysis	Rectal cancer	Robotic vs Laparoscopic	Robotic showed lower conversion, reoperation, and CRM positivity rates.	High
Eltair et al.(2020)	Meta-analysis	Total mesorectal excision	Robotic vs Laparoscopic	Comparable outcomes overall; robotic had longer operative time and shorter time to diet.	Moderate
Han et al.(2020)	Meta-analysis	Proctectomy	Robotic vs Laparoscopic	Robotic had longer operation time but lower conversion and erectile dysfunction rates.	Moderate
Chen et al.(2024)	Meta-analysis	Pelvic lymph node dissection	Robotic vs Laparoscopic	Robotic harvested more nodes and caused fewer complications, especially urinary retention.	High
Ryan et al.(2021)	Network meta-analysis	Total mesorectal excision	Robotic vs Laparoscopic vs Open vs Transanal	No long-term differences; robotic improved recovery but had longer OR time and higher cost.	Critically Low
Yang et al.(2023)	Meta-analysis	Colorectal cancer	Robotic vs Laparoscopic	Similar outcomes; robotic had longer surgeries and higher costs.	High
Villafane Asmat et al.(2024)	Systematic review	Rectal cancer surgery	Robotic vs Laparoscopic	No significant differences in complications, mortality, and leakage rates.	High
Zhu et al.(2024)	Systematic review	Rectal cancer surgery	Robotic vs Laparoscopic	Robotic surgery showed better urinary and sexual function preservation.	High
Ohtani et al.(2018)	Meta-analysis	Rectal cancer surgery	Robotic vs	Robotic surgery had lower conversion	Critically Low

			Laparoscopic	rates but longer operative time.	
Milone et al.(2019)	Meta-analysis	Total mesorectal excision	Robotic vs Laparoscopic	Robotic surgery yielded higher rate of complete mesorectum excision.	High
Zhu et al.(2021)	Meta-analysis	Right colectomy	Robotic vs Laparoscopic	Robotic colectomy showed less blood loss and fewer conversions.	Critically Low
Tang et al.(2018)	Meta-analysis	Rectal cancer surgery	Robotic vs Laparoscopic	Robotic surgery improved early postoperative urogenital function.	Low
Falola et al.(2025)	Systematic review	Colorectal cancer surgery	Robotic vs Laparoscopic	Robotic surgery had longer time but lower conversion and morbidity rates.	High
Gavriilidis et al.(2020)	Meta-analysis	Total mesorectal excision	Robotic vs Laparoscopic	Robotic surgery had lower conversion rate; other outcomes were similar.	Low
Ricciardi et al.(2025)	Systematic review	Colorectal oncologic surgery	Robotic vs Laparoscopic vs Open	Robotic surgery reduced complications, transfusions, and mortality.	High
Geitenbeek et al.(2024)	Systematic review	Total mesorectal excision	Robotic vs Laparoscopic vs Open vs TaTME	No significant functional or QoL differences; better urinary function at 3 months with robotics.	High
de'Angelis et al.(2025)	Meta-analysis	Rectal cancer resection	Robotic vs Laparoscopic vs Open	Robotic and transanal approaches yield better mesorectal excision and lower CRM positivity.	High
Huang et al.(2019)	Systematic review	Mesorectal excision	Robotic vs Laparoscopic	Robotic surgery lowers conversion rate; similar pathology outcomes to laparoscopy.	Low
Thrikandiyur et al.(2024)	Systematic review	Colorectal surgery	Robotic vs Laparoscopic	Fewer conversions and improving outcomes over time with robotic surgery.	High
Slim et al.(2024)	Meta-analysis	Mid-low rectal cancer	Robotic vs Laparoscopic	Robotic surgery has lower conversion rate; longer operative time.	Moderate
Yang et al.(2024)	Meta-analysis	Rectal cancer surgery	Robotic vs Laparoscopic	Robotic surgery improves urinary and sexual function recovery.	Low
Ishizuka et al.(2024)	Systematic review	Mesorectal excision	Robotic vs Laparoscopic	Robotic surgery decreases CRM positivity risk.	Moderate
Chen et al.(2024)	Systematic review	Colorectal surgery in obese patients	Robotic vs Laparoscopic	Robotic approach reduces blood loss and hospital stay, but increases operative time.	Low

Chaouch et al.(2024)	Systematic review	TME with LPND for rectal cancer	Robotic vs Laparoscopic	Robotic approach reduces urinary complications and increases lymph node yield.	Moderate
Phan et al.(2019)	Meta-analysis	Rectal cancer surgery	Robotic vs Laparoscopic	Robotic surgery significantly reduces conversion to open surgery.	Low
Stylianidi et al.(2024)	Meta-analysis	Single-port colorectal surgery	Robotic vs Laparoscopic	Similar outcomes; robotic approach yields more lymph nodes in oncologic cases.	Low
Kossenas et al.(2025)	Systematic review	Right colectomy	Robotic vs Laparoscopic	Robotic surgery had fewer complications but longer operative time in elderly patients.	Moderate
Liao et al.(2025)	Meta-analysis	Right colectomy with IA/EA	Robotic vs Laparoscopic vs Open	Robotic intracorporeal anastomosis reduced blood loss, ileus, and hospital stay.	Low
Bao et al.(2021)	Meta-analysis	Ventral mesh rectopexy	Robotic vs Laparoscopic	Robotic rectopexy had fewer complications and shorter hospital stay but longer operation time.	Low
Liu et al.(2021)	Meta-analysis	Rectal cancer surgery	Robotic vs Laparoscopic	No significant differences in complications; both approaches equally safe.	Low
Zhu et al.(2025)	Meta-analysis	Mid-low rectal cancer post-NCRT	Robotic vs Laparoscopic	Robotic surgery improved TME rate and recovery; had longer operative time.	Moderate
Shen et al.(2024)	Meta-analysis	LLND for rectal cancer	Robotic vs Laparoscopic vs Open	Robotic approach minimized complications and hospital stay; longest operative time.	Low
Wang et al.(2024)	Meta-analysis	NOSE for colorectal tumors	Robotic vs Laparoscopic	Robotic NOSE reduced blood loss and ileus with similar overall complications.	Moderate
Shi et al.(2024)	Meta-analysis	LLND for rectal cancer	Robotic vs Laparoscopic	Robotic LLND had lower complications and shorter stay; longer operative time.	Moderate
